# Chemical Profiling and Scaffold-Based Drug-Discovery Analysis of Bioactive Compounds from *Ceratonia siliqua* L. with Computational and Biological Validation

**DOI:** 10.1021/acs.jcim.6c00748

**Published:** 2026-05-28

**Authors:** Deli-Bright N. T. Oku, Garland Kgosi More, Yannick Nuapia, Ramakwala Christinah Chokwe

**Affiliations:** † Department of Chemistry, The Science Campus, College of Science Engineering and Technology, 219924 University of South Africa, Corner Christiaan de Wet Road and Pioneer Avenue, Florida Park, Roodepoort 1709, South Africa; ‡ College of Agriculture and Environmental Sciences, CAES Laboratories, University of South Africa, Private Bag X6, Johannesburg 1710, Florida, South Africa; § Department of Pharmacy, Turfloop Campus, School of Healthcare Science, 37714 University of Limpopo, Polokwane 0727, South Africa

## Abstract

The scaffold concept is central in medicinal chemistry and drug design to generate, analyze, and capture the core structural frameworks that define bioactive compounds. Natural products and their underlying molecular scaffolds have long provided many of the biologically active ingredients in modern medicines and continue to inspire new therapeutic agents. However, linking these core structural frameworks to observed biological activity remains a key challenge in natural-product research. Here, this study integrates a computational–experimental approach combining scaffold-based drug analysis, chemical profiling, and computational and biological validation to identify bioactive motifs in *Ceratonia siliqua* L pods. A total of 253 identified compounds from the gas chromatography–mass spectrometry (GC–MS) profiling were clustered and grouped into antioxidant, antimicrobial, and cytotoxic activity sets, after which their Bemis–Murcko scaffolds were extracted using RDKit. The most common scaffolds were ranked and visualized to give a clear picture of the prevalent structural patterns throughout the data sets. Experimental assays validated the computational dominant scaffold predictions, revealing that the antioxidant activity was associated with phenolic and terpenoid scaffolds, the antimicrobial response was aligned with monoterpenoid and cyclohexane/cyclohexene-based scaffolds, and the cytotoxic trends were consistent with small heterocycles and imide-containing scaffolds. To further bridge the gap between the identified scaffolds and their corresponding biological activity, molecular docking was performed against key biological targets, including KEAP1, *Staphylococcus aureus* DHFR, and EGFR. The docking results demonstrated favorable binding interactions and identified key ligand–protein interactions, supporting the potential contribution of the GC–MS-identified compounds to the observed biological activities of the plant extract. These scaffold–activity relationships demonstrate that recurring structural motifs are associated with the biological effects observed in *C. siliqua* L., highlighting the plant as a promising source of pharmacologically relevant scaffolds for drug discovery.

## Graphical abstract

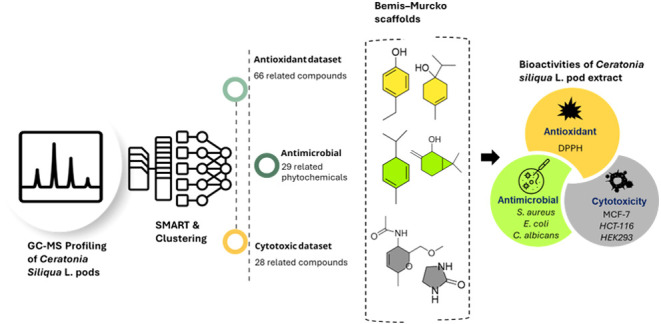

## 1 Introduction

Medicinal plants, including *Ceratonia siliqua* L., remain chemically rich sources of bioactive compounds and distinctive molecular scaffolds with potential relevance to treatment of various diseases.^1,2^ Their structural variety, chemical diversity, evolutionary optimization, and extensive bioactivity provide unique scaffolds capable of engaging biological targets with high specificity and potency.^3,4^ Bioactivity-guided investigations of plant extracts play a central role in modern drug-discovery research, as they help identify extracts or compounds with pharmacological potential.^5^ When these biological activities are integrated with scaffold-based analysis, the approach enables the elucidation of the core structural motifs that may underlie the observed biological effects.^6^

Natural products, particularly those derived from medicinal plants, have been the source of most of the biologically active ingredients of medicines and continue to serve as templates for new therapeutic agents.^7,8^ A major challenge in natural-product research is establishing a clear connection between the chemical constituents of an extract and their biological activities. Analytical techniques, including GC–MS and LC–MS, can detect a variety of plant metabolites; however, various studies describe the chemical profile of plant extracts without directly connecting their structural frameworks to the observed biological activities.^9–13^ Such disconnect hampers the elucidation of features responsible for specific bioactivities due to their structural components in a mixture.

Scaffold-based drug discovery represents an effective framework that has the potential to bridge this gap. Scaffolds are defined by the core ring systems and linker frameworks that form the central architecture of a compound, are strongly associated with biological behavior, and are key determinants of drug-likeness.^14^ Natural-product scaffolds are widely regarded as “privileged structures” because of their ability to be the basis for successful drugs.^15^ These scaffolds are being used as cores of compound libraries, including those generated through combinatorial techniques. These libraries are inspired by major natural-product classes such as alkaloids, polyketides, terpenoids, and flavonoids, and they demonstrate how these scaffolds can expand chemical space and improve the likelihood of discovering biologically active molecules.^16^ Building on this principle, researchers can compare the chemical constituents of plant extracts by reducing complex molecular structures to their fundamental scaffolds, allowing the identification of recurring structural patterns within bioactive extracts and enabling the inference of preliminary structure–activity relationships.^17^ This method has been successfully applied in medicinal chemistry and virtual screening studies yet remains underutilized in natural products research where chemically rich plant extracts offer numerous unexplored scaffolds.^18^ This scaffold-based approach is particularly valuable for plant extracts that contain both volatile and nonvolatile compounds, where multiple chemical classes may synergistically or independently contribute to the observed biological activities.^19^ With the application of various techniques to create analogues and derivatives of plant-based natural products, it becomes possible to derive novel compounds that can be patented, even when the original structure was previously disclosed. Plant-derived natural products from *C. siliqua* L. (carob tree), a Mediterranean species,^20^ are well-known for their antioxidant,^21^ anti-inflammatory,^22^ and antimicrobial properties.^23,24^ The plant is also a rich source of valuable bioactive compounds used across cosmetics, agriculture, and pharmaceutical industries.^25^ Therefore, integrating scaffold analysis of carob pod compounds with bioassays and in silico evaluation strengthens the discovery process by pinpointing the key structural groups responsible for different biological activities observed in the extract.

In this study, hexane extract of *C. siliqua* L. pods was prepared using Soxhlet extraction, which was subsequently subjected to gas chromatography–mass spectrometry analysis to determine their chemical composition. The antioxidants, antimicrobial, and cytotoxic activities of the extract were evaluated, and the identified compounds from GC–MS were further examined through scaffold-based drug-discovery analysis to determine the core structural frameworks associated with each biological effect. Overall, the study aims to uncover structural scaffolds, clarify structure–activity relationships, and explore the broader drug-discovery potential of *C. siliqua* L.

## 2 Materials and Methods

### 2.1 Plant Material

Fresh pods of *C. siliqua* L. (carob pods) were sampled from Lindhaven, Roodeport, in the Gauteng province of South Africa (26.1458° S, 27.8502° E), in October 2025. The samples were transported to the laboratory at the Department of Chemistry, University of South Africa (UNISA), Florida Campus, and stored in a refrigerator at 3 °C. Plant identification and authentication were carried out at the College of Agriculture and Environmental Sciences Horticulture Research Centre, UNISA. The specimens of the carob pod (UNISA/CAES/2025/SS0028) were deposited at the University Herbarium. The plant specimens were air-dried under direct sunlight for 14 days. The dehydrated pods were ground together to a fine powder using a high-speed pulverizette 19 cutting mill (FRITSCH GmbH, Germany), which was immediately used for further analysis.

### 2.2 Extraction of Phytochemicals

Approximately 40 g of ground *C. siliqua* pods were weighed and packed into a thimble, which was placed in a 250 mL Soxhlet extractor for extraction. The material was extracted with 300 mL of hexane (1084 BATCH 24703313) under reflux at 50–60 °C for 6 h. After extraction, the mixture was filtered through Whatman No. 1 filter paper, and the filtrate was concentrated using a Stuard RE300 rotary evaporator (Keison Products, U.K.) to concentrate the extract. The resulting crude extract was further allowed to dry at room temperature on a sample vial and subsequently stored at −20 °C until further use.

### 2.3 Chemical Composition Analysis

#### 2.3.1 Gas Chromatography–Mass Spectrometry (GC–MS) Analysis

The hexane extract of the carob pod was filtered using a 0.45 μm PVDF membrane filter prior to analysis. The chemical constituents of the filtered *C. siliqua* L. hexane extract were then analyzed using a gas chromatography–mass spectrometry (GC–MS) system. The analysis was performed on a LECO Pegasus 4D GC HRT-MS (high-resolution time-of-flight mass spectrometer) with a modulator and a secondary oven (LECO Corporation, St Joseph, USA), equipped with an Agilent 7890A gas chromatograph (Agilent Technologies, Inc., Wilmington, DE, USA). The GC capillary column Rxi-5Sil MS (30 m × 0.25 mm i.d., 0.25 μm film thickness) was used for the separation of volatile compounds, with helium as the carrier gas at a constant flow rate of 1.0 mL/min. A 1 μL aliquot of the extract was injected into a splitless mode for 60 s, and the injector temperature was set at 220 °C.

The gas chromatograph program began at 38 °C and was held for 3 min, followed by a gradual increase at 12 °C/min until the oven temperature reached 180 °C (held for 5 min), then ramped at 40 °C/min to 220 °C and held for 2 min. The transfer-line and ion-source temperatures were set at 230 and 180 °C, respectively, and the total run time was 24.5 min. Mass spectrometric detection was performed under electron-impact ionization (70 eV) with a scan range of *m*/*z* 40–500. Retention time, peak detection, and peak matching were performed using ChromaTOF software (LECO, St. Joseph, USA).^26^ Finally, peaks identification was performed by comparing the spectra with those listed in the RepLib, MainLip, and NIST Retention Index (NIST_RI) mass spectral database.

### 2.4 Scaffold-Based Drug-Discovery Analysis

#### 2.4.1 Compound Preparation and Molecular Fingerprinting

All compounds identified from GC–MS analysis were first standardized and mapped to their respective simplified molecular-input line-entry system (SMILES) and PubChem CIDs using the PubChemPy Python package. Each compound name was queried against PubChem to retrieve its canonical SMILES and unique PubChem CIDs. Compounds that could not be found were recorded and excluded from further processing.

After retrieving SMILES, molecules were converted to RDKit molecule objects, and molecular fingerprints were generated using the extended-connectivity fingerprint (ECFP4) with a radius of 2 and a bit vector length of 1024.^27^

#### 2.4.2 Compound Space Network Construction

A compound space network (CSN) was constructed to explore the structural relationships among all GC–MS-derived compounds. In this network, each compound represents a node, and edges were created between pairs of compounds with a Tanimoto similarity greater than 0.6 based on ECFP4 fingerprints. Structural clusters were identified using the greedy modularity optimization algorithm in NetworkX.

For each cluster using eq 1, homogeneity was calculated as the ratio between the average pairwise Jaccard distance of compounds within the cluster (intracluster distance) (eq 2) and the average distance between compounds inside the cluster and all compounds outside the cluster (intercluster distance) (eq 3). Mathematically, this metric is defined as

1 $$h\mathrm{omogeneity}\;\left(C_{i}\right)=\frac{a\mathrm{verage intra}‐\mathrm{cluster distance}}{a\mathrm{verage inter}‐\mathrm{cluster distance}}$$

2 $$i\mathrm{ntra}‐\mathrm{cluster distance}=\frac{1}{{n_{i}}^{2}}\sum_{x\in C_{i}}\sum_{y\in C_{i}}\mathrm{dis}\mathrm{tance}\left(x,y\right)$$

3 $$\mathrm{inter}‐\mathrm{cluster distance}=\frac{1}{n_{i}\cdot N}\sum_{x\in C_{i}}\sum_{y\in C_{i}}\mathrm{distance}\left(x,y\right)$$

where *n_i_* is the number of items in cluster *C_i_*.

#### 2.4.3 Activity-Based Data Set Construction

Activity-based data sets were constructed by first identifying molecules as antioxidant, antimicrobial, or cytotoxic using SMARTS-defined (SMILES Arbitrary Target Specification) substructures derived from pharmacophore patterns reported in the literature. These SMARTS rules, originally defined by Daylight Chemical Information Systems,^28^ were implemented in this study using the RDKit cheminformatics toolkit. Antioxidant compounds were recognized through motifs such as phenols, catechols, and hydroquinone-like structures, as described by Rice-Evans et al. and Pietta (2000),^29–31^ antimicrobial compounds were assigned using patterns that matched imidazoles, purine-like heterocycles, chlorinated aromatics, and terpenoid-related structures, following the descriptions of Nguyen (2022)^32–34^ based on the research of Ghods et al., and the cytotoxic compounds were also identified by the presence of quinones, amides, and epoxide or strained-ring motifs.^35^

In the construction of this activity-based data set, each molecule that matched one of these SMARTS patterns was classified as a proof compound for that specific activity. To capture structural diversity beyond these direct matches, each data set was then expanded to include all compounds within the clusters containing at least one proof compound. This produced activity-informed and cluster-expanded data sets for the antioxidant, antimicrobial, and cytotoxic compound groups. The expanded data sets combined pharmacophore-guided selection with cluster-based structural improvement, which was later used for scaffold analysis to identify the fundamental structural frameworks associated with each biological activity.

#### 2.4.4 Scaffold Analysis of GC–MS Activity-Based Compounds

Scaffold analysis was carried out in Python using RDKit (https://www.rdkit.org/) for the constructed antioxidant, antimicrobial, and cytotoxicity data sets. All SMILES strings from the various GC–MS activity-based data sets were run through a custom sanitization function (safe_mol) to ensure that only valid molecules were present for the scaffold-based drug analysis. For each present valid molecule, Bemis–Murcko (BM) scaffolds and generalized cyclic skeletons were extracted using RDKit’s MurckoScaffold.GetScaffoldForMol and MurckoScaffold.MakeScaffoldGeneric, respectively.^36,37^ The scaffold sets that were produced from the three activity-based data sets were used to compute several data set level metrics. Among these were the total number of molecules (*N*), the number of unique BM scaffolds (*N*_s_), the number of singleton scaffolds appearing only once (*N*_ss_), and the number of unique cyclic skeletons (*N*_csk_). Scaffold proportions relative to data set size were computed as $\frac{N_{s}}{N},\frac{N_{\mathrm{ss}}}{N},\frac{N_{\mathrm{csk}}}{N}$ . Scaffold diversity was calculated following the definitions used in previous studies: Scaffold diversity = $\frac{N}{M}$ and Singleton diversity = $\frac{N_{\mathrm{sing}}}{M}$ , where *N* is the number of unique scaffolds, *N*_sing_ is the number of singleton scaffolds, and *M* is the total number of molecules. Shannon entropy (SE) based on scaffold frequency distribution was also calculated using eq 4

4 $$\mathrm{SE}=-\sum_{i=1}^{n}{p_{i}\log}_{2}\left(p_{i}\right)$$

where $p_{i}=\frac{C_{i}}{P}$ , *C*_*i*_ is the count of each scaffold, *P* is the total number of scaffold assignments, and *n* is the number of unique scaffolds.

Lastly, the frequency of the core structural motifs was used to rank the top dominant scaffolds in each data set. The drawing functions in Matplotlib, Seaborn, and RDKit were used to generate bar plots of the top ten scaffolds and grid images of the related structures. These visuals, together with the Bemis–Murcko scaffold generation, cyclic skeleton extraction, and scaffold-level calculations, provided a clear overview of the dominant structural patterns across the antioxidant, antimicrobial, and cytotoxic compounds, forming a consistent and reproducible pipeline for assessing scaffold diversity in the GC–MS-derived compounds of *C. siliqua* L.

### 2.5 Molecular Docking of GC–MS-Identified Bioactive Scaffold-Derived Compounds

The integration of molecular docking simulations provides a mechanistic insight into the potential bioactivity of the GC–MS-identified compounds from the identified scaffolds, allowing assessment of binding affinity, molecular interactions, and the potential contribution of individual compounds to the observed antioxidant, antimicrobial, and cytotoxic activities. The identification of potent ligands against representative biological targets is critical for analyzing the structure–activity relationship responsible for the biological effects observed in *C. siliqua* L pod extract. Representative biological targets were selected based on relevance to the observed assays: Kelch-like ECH-associated protein 1 (KEAP1) for antioxidant response (PDB: 5WG1),^38^ *Staphylococcus aureus* dihydrofolate reductase (DHFR) (PDB: 3FRD) for antimicrobial activity,^39^ and EGFR (PDB: 7ZYM) for cytotoxicity in ER-positive breast cancer (MCF-7).^40^ Their crystal structures were downloaded from the RCSB Protein Data Bank^41^ and prepared using Discovery Studio Visualizer (v19.1.0.18287) by removing heteroatoms and water molecules, followed by energy minimization prior to docking. Molecular docking simulations were performed using AutoDock Vina within PyRx, with grid parameters centered on the reported binding pockets of each receptor in the literature. Selected ligands, including GC–MS-identified constituents from the bioactive scaffolds and reference standard drugs for each bioactivity (ascorbic acid, chlorhexidine, gentamicin, and doxorubicin), were energy-minimized using the MM2 force field in Chem3D (PerkinElmer Informatics, 2020) and converted to PDB format for docking. Docking outputs were analyzed using RMSD docking scores, and ligand–protein interactions were visualized with Discovery Studio for 2D interaction maps.

### 2.6 Antioxidant Activity of *C. siliqua* L. Pod Extract

#### 2.6.1 2,2-Diphenyl-1-picrylhydrazyl (DPPH) Free-Radical-Scavenging (RSA) Assay

In order to access the antioxidant activity of the hexane extract of *C. siliquia* L pods, the free-radical-scavenging activity of the extract was evaluated using the 2,2-diphenyl-1-picrylhydrazyl (DPPH) assay, according to a reported method.^42^ In this regard, 20 μL samples were mixed with 180 μL of methanol and 100 μL of DPPH at a concentration of 0.1 mM under low light. A microplate reader (Varioskan Flash, Thermo Fisher Scientific, Finland) was used to measure the absorbance of the resultant mixture at 517 nm after 30 min of incubation at 25 °C in the dark. Ascorbic acid served as the positive control. The samples were analyzed in sets of three, and the experiment was conducted two times to confirm consistency. Each extract’s %RSA or positive control’s percentage RSA was calculated using eq 5

5 $$\mathrm{RSA}\left(\%\right)=100\times \left(1-\frac{\mathrm{AE}}{\mathrm{AD}}\right)$$

where AE denotes the absorbance of the mixture of the plant extract or ascorbic acid, while AD represents the absorbance of DPPH without the extract or ascorbic acid.

### 2.7 Antimicrobial Activity of *C. siliqua* L. Pod Extract

Three microorganisms were utilized in this investigation, consisting of two bacterial strains *Escherichia coli* (ATCC 8739) (Gram-negative) and *S. aureus* (ATCC 11632) (Gram-positive), and a fungus *Candida albicans* (ATCC 10231), which were obtained from Anatech Instruments (Pty) Ltd., located in Randburg, Gauteng, South Africa (Anatech, Kwik-stick). Bacterial strains were cultivated in nutrient agar, whereas the fungus was grown on Sabouraud Dextrose Agar (SDA) and incubated for 24 h at 37 °C under aerobic conditions. Pure colonies of the microorganisms from the agar were transferred into nutrient broth and Sabouraud dextrose broth (SDB), respectively, to create an overnight culture. After 24 h, the cultures were centrifuged for 10 min to obtain a microbial pallet, and fresh media was added after the old media was aspirated.

Antimicrobial activity was assessed using the microplate serial dilution method as described by Asong et al.^43^ The minimum inhibitory concentration (MIC) was defined as the lowest concentration of the substance that can inhibit microbial growth. The microorganisms were adjusted to match the 0.5 McFarland standard solution using a spectrophotometer; the microbial cell density at an optical density of 600 nm was approximately 0.08–0.13, resulting in a concentration of 1.5 × 10^8^ CFU/mL in sterile 0.85% sodium chloride (NaCl) solution. The extract was assessed at concentrations ranging from 39.06 to 5000 μg/mL in 96-well plates and was incubated with microorganisms for 24 h at 37 °C. Gentamicin and chlorhexidine served as positive controls for the bacterial and fungal assays, respectively. Furthermore, sterile distilled 5% DMSO functioned as a negative control. A staining reagent, 50 μL of Resazurin sodium salt at a concentration of 0.2 mg/mL, was used as an indicator to assess microbial growth. The minimum inhibitory concentration (MIC) was determined through visual observation following the Clinical and Laboratory Standards Institute (CLSI) reference method (Approved Standard M27-A3, NCCLS, Wayne, PA, USA).^44^

### 2.8 Cytotoxicity Activity of *C. siliqua* L. Pod Extract

The MTT (3-[4,5-dimethylthiazol-2-yl]–2,5-diphenyl tetrazolium bromide) cell proliferation assay was employed to evaluate the cytotoxic effects of the extracts.^43,45^ Human embryonic kidney (Hek293), breast cancer (MCF-7), and colorectal cancer (HCT-116) cell lines were cultured in Dulbecco’s modified Eagle’s medium (DMEM, Gibco) that included 10% fetal bovine serum (FBS) and 1% penicillin/streptomycin in culture flasks maintained at 37 °C with 5% CO_2_. Cells were seeded at a density of 1 × 10^4^ cells per 100 μL in 96-well plates and left to incubate overnight. After this initial treatment, the cells were further incubated for a full day at varying concentrations between 100 and 0.39 μg/mL using a series of diluted extracts and doxorubicin, followed by an additional 4 h incubation at 37 °C. Doxorubicin was used alongside DMSO (1%), with untreated cells acting as controls. To ensure that any cytotoxic effects observed were due solely to the compounds and not the DMSO, the final concentration of DMSO was maintained at 0.1% (v/v) in all experimental wells. Finally, 20 μL of MTT was added to each well in a microtiter plate, and 100 μL of DMSO was introduced to dissolve the MTT. Cell viability was assessed utilizing the following equation (eq 6)­

6 $$\left(\%\right)\;c\mathrm{ell viability}=\left(\frac{A_{t}}{A_{b}}\right)\times 100$$

where *A*_t_ represents the absorbance of the treated cells, and *A*_b_ indicates the absorbance of control cells. Cytotoxicity was expressed as the 50% inhibitory concentration (IC_50_) of the tested plant extracts that caused a 50% reduction in viable cells relative to the control cells. The data are presented as the mean ± standard deviation (SD) from a minimum of three independent experiments (*p* < 0.0001). Comparisons among the compounds and the positive control (doxorubicin) were conducted using one-way analysis of variance (ANOVA). To identify particular differences between the means, Tukey’s significant difference test was utilized.

### 2.9 H2DCF-DA Reactive Oxygen Species (ROS) Detection Assay

Cell-based detection of ROS was investigated utilizing a 2′,7′-dichlorodihydrofluorescein diacetate (H2DCF-DA) assay in Hek293-T, HCT-116, and MCF-7 macrophage cells, following the method by More et al.^46^ Cells were seeded at 6 × 10^4^ cells/well in a 96-well plate and incubated for 24 h at 37 °C in a humidified atmosphere containing 5% CO_2_. After incubation, the cells were treated with the *C. siliqua* pod extract at a concentration of 100 μg/mL. Lipopolysaccharide (LPS, 1 μg/mL) was used as a positive control to induce ROS production. Following over 24 h of incubation and treatment, 10 μM of H2DCF-DA was added for 30 min in the dark. The fluorescence was measured using a microplate reader at 485 and 535 nm excitation and emission, respectively.

## 3 Results and Discussion

### 3.1 Gas Chromatography–Mass Spectrometry (GC–MS) Analysis

The GC–MS analysis of the hexane extract of *C. siliqua* L. showed the pods contain a wide variety of phytochemical constituents. The identified compounds were distributed across several chemical groups, which included fatty acids, carboxylic acids, alcohols, amides, alkanes, esters, ketones, phenolic and aromatic compounds, aldehydes, as well as furan and pyran derivatives. Among these, fatty acids and carboxylic acids were the most prominent, contributing approximately 59.62% of the total composition, followed by alcohols (∼12.21%), amides (∼7.15%), alkanes (∼6.35%), and esters (∼4.32%), while all remaining classes together accounted for less than 6% of the total composition.

The total ion chromatogram (TIC) of the carob pod extract is shown in Figure 1. The identified compounds exhibited retention times ranging from 7.0329 to 24.222 min, with area percentages varying from 0.00198% to 16.0455%, reflecting differences in their relative abundances. Trans-13-octadecenoic acid was the most abundant constituent (16.05%), followed by *n*-hexadecanoic acid (7.58%), phenylethyl alcohol (7.39%), hexanoic acid (7.14%), and hexadecanoic acid, ethyl ester (5.52%).

**Figure 1:**
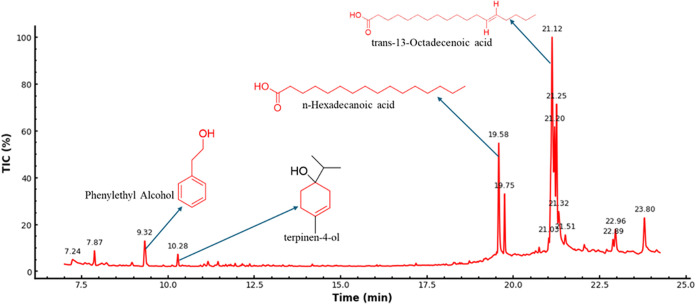
Total ion chromatogram (TIC) of the compounds obtained from the GC–MS run of the hexane extract from *C. siliqua* pods. Compound identities were assigned by matching their mass spectra with entries in the RepLib, MainLip, and NIST Retention Index (NIST_RI) mass spectral libraries.

Previous studies have reported on the biological activities of some of the compounds identified in this study. These included terpinen-4-ol (1.29%), d-limonene (0.28%), benzyl alcohol (0.51%), benzeneacetaldehyde (0.58%), 1,2-hydrazinedicarboxaldehyde (0.09%), phenol, 4-ethyl-2-methoxy- (0.20%), ethanol, 2-phenoxy- (0.10%), 3-phenylpropanol (0.40%), and l-glutamine *tert*-butyl ester (0.09%). Many of these compounds are associated with antimicrobial, antioxidant, anti-inflammatory, and anticancer properties, while others, such as l-glutamine *tert*-butyl ester and certain propanoic acid derivatives, may be involved in metabolic regulation. A significant number of detected compounds that were present had no previously reported biological activities, suggesting the need for further investigation of their pharmacological potential.

### 3.2 Compound Clustering and Structural Cohesion

The 253 compounds that were identified from the GC–MS analysis were distributed across all clusters that were generated using ECFP4 fingerprints and greedy modularity optimization (Figure 2). A total of 172 clusters were generated during the analysis. The cluster with ID number 0 contained the highest number of compounds (19), followed by Cluster ID 1 with 15 compounds and Cluster ID 2 with 7 compounds. The other clusters (that is, Cluster IDs 3–22) contain between 2 and 5 compounds, showing that the majority of clusters are relatively small. Focusing on clusters with five or more compounds highlighted 7 clusters comprising 61 compounds (24.1% of the data set) and represent the most structurally dense groups within the network.

**Figure 2:**
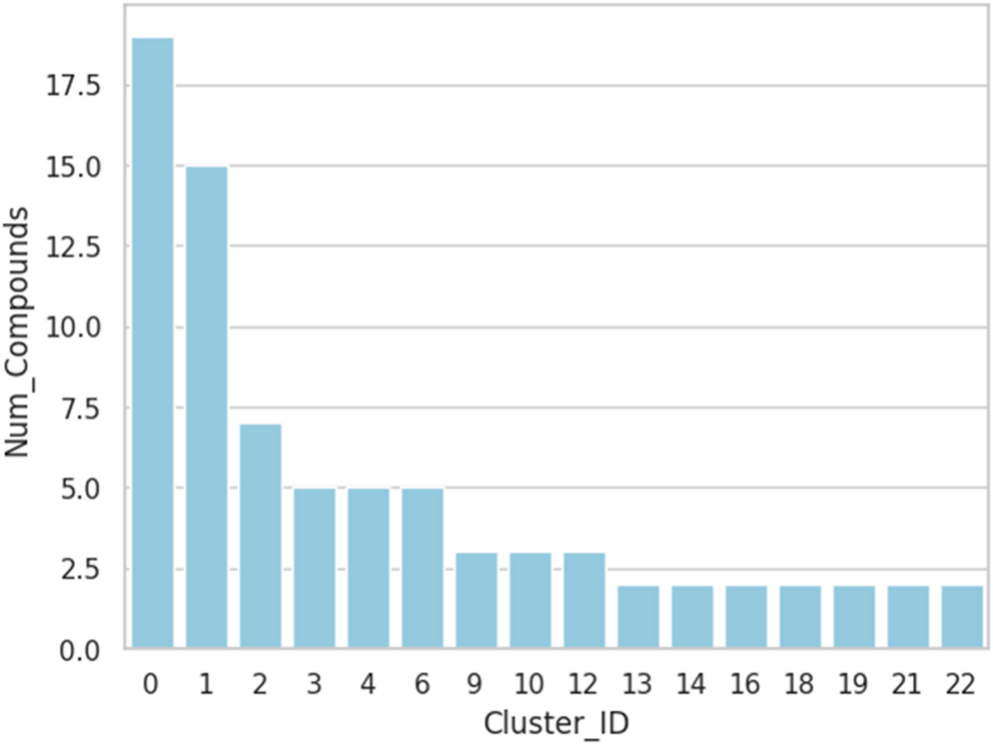
Distribution of GC–MS-identified compounds across clusters generated using ECFP4 fingerprints and greedy modularity optimization, showing the number of compounds assigned to each Cluster ID.

The clusters displayed a significant difference in their structural cohesion. Their homogeneity scores spanned a wide range from as low as 3.24 in Cluster ID 2 (7 compounds) to as high as 26.38 in Cluster 9 (3 compounds) (Figure 3). Several smaller clusters, including Cluster IDs 22, 19, 6, 4, 23, 9, 24, 10, 12, and 21, exhibited perfect intracluster similarity (1.0), indicating highly conserved core scaffolds (Figure 4). On the other hand, the largest clusters, like Cluster ID 0 (19 compounds; intrasimilarity = 0.595; homogeneity = 3.96) and Cluster ID 1 (15 compounds; intrasimilarity = 0.561; homogeneity = 3.60), had moderate similarity, indicating shared core structural frameworks with varied substituents (Table S1).

**Figure 3:**
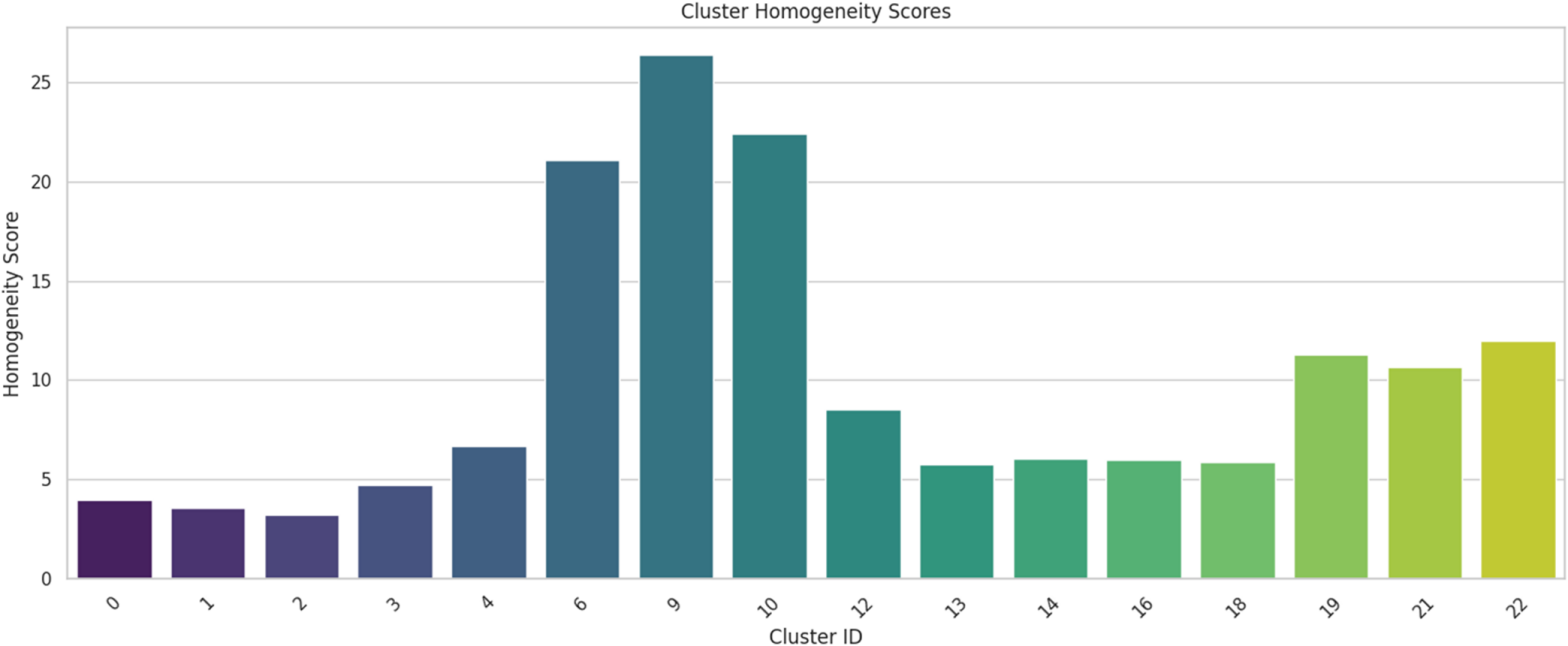
Homogeneity scores of GC–MS-derived clusters, showing variation in structural cohesion across Cluster IDs.

**Figure 4:**
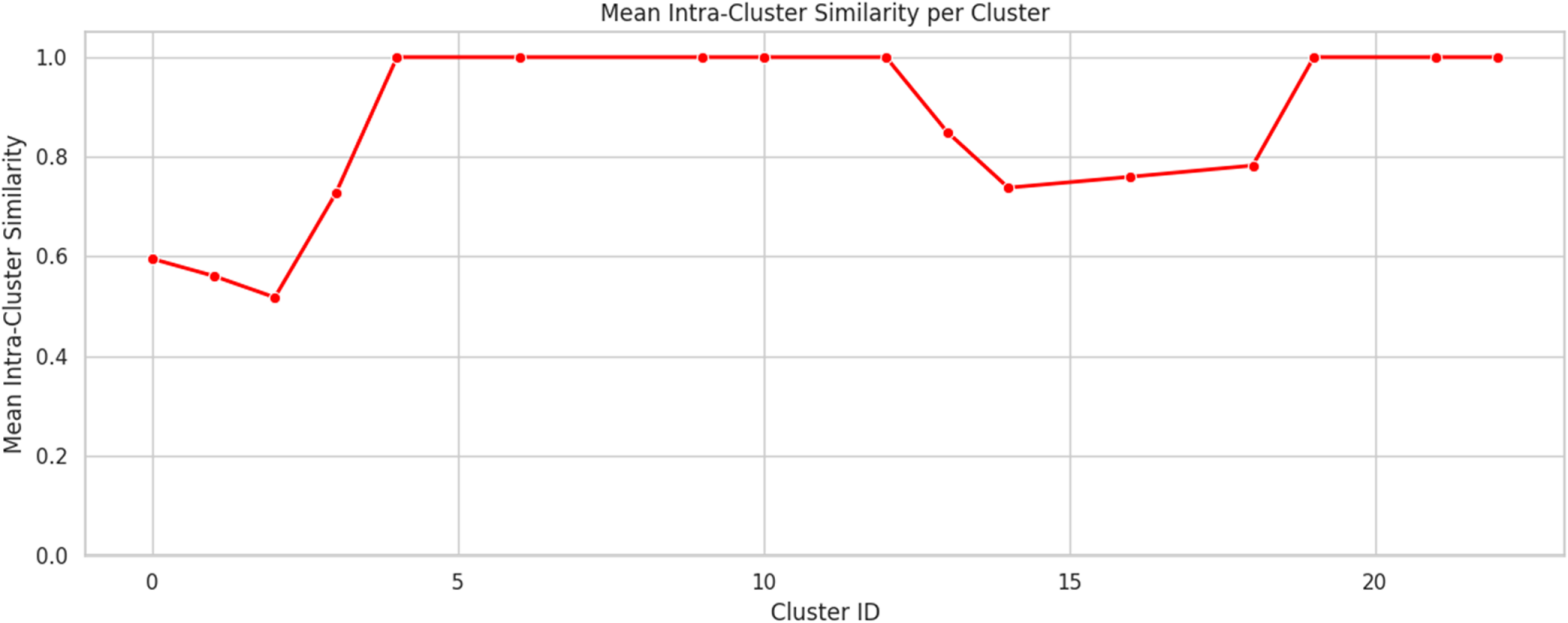
Mean intracluster similarity across all clusters, illustrating the degree of structural conservation within each cluster.

From Figure 5, a clear inverse trend between cluster size, intracluster Tanimoto similarity, and homogeneity for the GC–MS-identified compounds is visible. That is, smaller clusters consistently exhibit higher intracluster similarity, which is either 1.0 or approaching 1.0, and correspondingly higher homogeneity scores, indicating strong structural cohesion and conserved scaffolds in the GC–MS data set. In contrast, larger clusters show lower intracluster similarity, typically ranging between 0.55 and 0.70, reflecting greater structural diversity and more variable substituent patterns. These results confirm that the clustering approach successfully captures the balance between structural conservation and diversity across the data sets. Clusters with high similarity represent tightly conserved scaffolds, while clusters with lower similarity reflect scaffold frameworks with flexible or varied substituent regions. This structural organization provides a reliable basis for downstream scaffold-based bioactivity investigations of antioxidant, antimicrobial, and cytotoxic activities.

**Figure 5:**
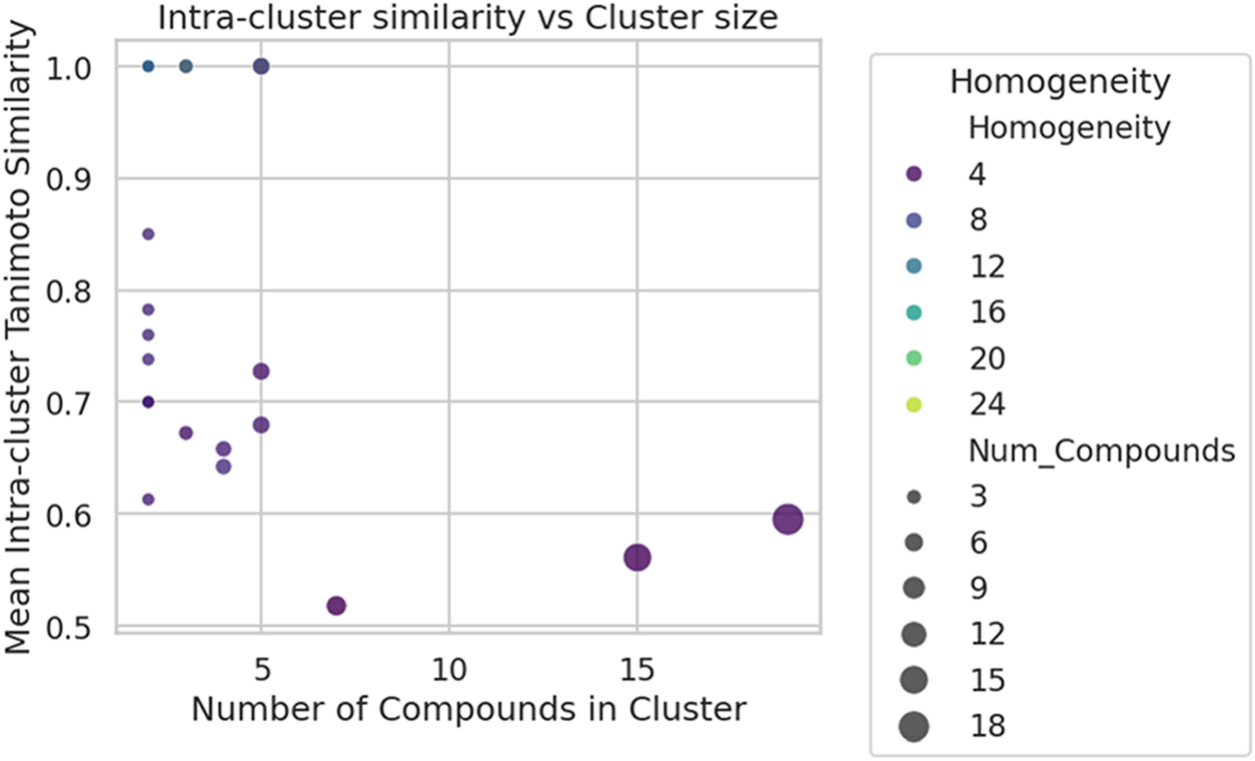
Relationship between cluster size, intracluster Tanimoto similarity, and homogeneity, showing that smaller clusters have higher similarity and homogeneity, while larger clusters exhibit greater structural diversity.

### 3.3 Activity-Driven Compound Selection and Data Set Expansion

The first phase of the activity-based data set construction was from the original GC–MS data set using literature-informed SMARTS patterns as proof-of-concept identifiers for each activity class. This detected 51 antioxidants, 29 antimicrobials, and 14 cytotoxic compounds, showing that antioxidant motifs were more common, while cytotoxic structures were scarce (Figure 6). These SMARTS patterns functioned as structural anchors, capturing characteristic motifs known to contribute to the respective bioactivities.

**Figure 6:**
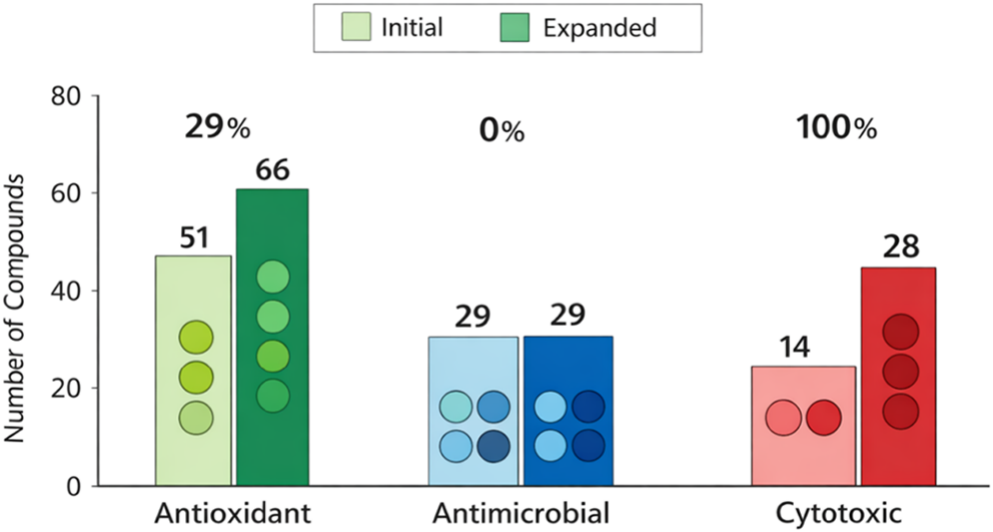
Bar chart representation of SMARTS-identified compounds and their expansion through structural similarity clustering across antioxidant, antimicrobial, and cytotoxic GC–MS data sets.

With the second phase, which is expanding the data sets using structural similarity clustering, the number of antioxidants increased to 66 and cytotoxics to 28, while antimicrobials remained at 29. This suggests that antioxidant and cytotoxic compounds share more conserved frameworks, while antimicrobial structures are more diverse (Figure 6). The expansion captured compounds beyond the initial SMARTS-defined motifs, enriching chemical diversity while remaining relevant to each bioactivity. This provides a stronger foundation for scaffold analysis and highlights trends in chemical space, with expansion ratios of ∼29% for antioxidants, 0% for antimicrobials, and ∼100% for cytotoxics, showing the approach is especially effective for underrepresented, structurally coherent compounds (Figure 6).

The resulting activity-based data sets provide a foundation for scaffold-based bioactivity exploration, enabling the identification of conserved core frameworks that may drive antioxidant, antimicrobial, and cytotoxic properties.

### 3.4 GC–MS Antioxidant Data Set

GC–MS profiling of *C. siliqua* L. identified a diverse set of phytochemical constituents, several of which are known or predicted to possess antioxidant activity. The antioxidant screening of the GC–MS data set, using literature-validated SMARTS substructures, highlighted a wide range of compounds containing phenolic motifs, benzylic alcohols, oxygenated monoterpenoids, aromatic aldehydes, and polyols, all of which are well-established contributors to free-radical quenching and redox modulation. Structural clustering (ECFP4; Tanimoto >0.6, allowing broader clustering and capture scaffold diversity) further expanded the antioxidant space by identifying compounds that share scaffold-level similarity with known antioxidant chemotypes even when they do not explicitly match the SMARTS rules. This expansion resulted in 66 antioxidant-related compounds in the final data set (Table 1).

**Table 1:** GC–MS-Identified Antioxidant Compounds Based on SMARTS Pharmacophores and Structural Clustering^a^

name	R.T. (min)	formula	peak S/N	base mass	area (%)	PubChem_CID	antioxidant (SMART)	cluster ID	antioxidant (cluster)
butanoic acid	7.032	C_4_H_8_O_2_	155	60.06	0.09	264	+	0	–
hexanoic acid	7.236	C_6_H_12_O_2_	3442	60.06	7.14	8892	+	0	–
1,3-dioxolane-2-methanol	7.243	C_4_H_8_O_3_	72	73.06	0.31	294864	+	27	–
benzyl alcohol	8.078	C_7_H_8_O	378	79.09	0.50	244	+	32	–
2-nonanone	8.714	C_9_H_18_O	246	58.08	0.13	13187	–	0	–
phenylethyl alcohol	9.324	C_8_H_10_O	7911	91.08	7.39	6054	+	15	–
terpinen-4-ol	10.282	C_10_H_18_O	1617	71.09	1.29	11230	+	53	–
2-heptanone	10.355	C_7_H_14_O	56	58.08	0.03	8051	–	0	–
phenol, 4-ethyl	10.411	C_8_H_10_O	340	107.0	0.19	31242	+	55	+
ethanol, 2-phenoxy-	11.072	C_8_H_10_O_2_	232	94.07	0.10	31236	+	17	+
3-phenylpropanol	11.152	C_9_H_12_O	570	91.08	0.39	31234	+	15	–
cyclohexanol, 1-ethenyl-	11.231	C_8_H_14_O	63	70.08	0.03	74,741	+	64	–
1,2,4-benzenetricarboxylic acid, 1,2-dimethyl ester	11.335	C_11_H_10_O_6_	17	207.0	0.04	610,016	+	65	–
2,6-octadiene-4,5-diol,3,6-dimethyl-	11.374	C_10_H_18_O_2_	321	85.06	0.12	5362856	+	67	–
2-methoxybenzyl alcohol	11.595	C_8_H_10_O_2_	99	77.07	0.03	69154	+	70	+
phenol, 4-ethyl-2-methoxy-	11.756	C_9_H_12_O_2_	518	137.1	0.20	62465	+	72	+
1,4-dihydroxy-*p*-menth-2-ene	12.099	C_10_H_18_O_2_	115	109.1	0.04	300085	+	76	–
*trans*-3(10)-caren-2-ol	12.149	C_10_H_16_O	249	71.09	0.15	572861	+	78	–
bicyclo[2.2.1]heptan-2-ol, 6-*tert*-butyl-	12.359	C_11_H_20_O	141	55.09	0.05	549167	+	80	–
2-methyl-5-(propan-2-ylidene)cyclohexane-1,4-diol	12.368	C_10_H_18_O_2_	393	74.07	0.19	91697160	+	81	–
1,3-diacetin	12.562	C_7_H_12_O_5_	46	103.0	0.02	66924	+	84	–
2,2,4-trimethyl-1,3-pentanediol diisobutyrate	12.748	C_16_H_30_O_4_	233	71.09	0.15	23284	–	20	–
dasycarpidol	12.789	C_17_H_22_N_2_O	15	105.1	0.00	576461	+	86	–
1,2-cyclohexanediol, 1-methyl-4-(1-methylethyl)-	12.860	C_10_H_20_O_2_	137	71.09	0.08	36574	+	87	–
propanoic acid, 2-methyl-, 3-hydroxy-2,2,4-trimethylpentyl ester	13.046	C_12_H_24_O_3_	223	71.09	0.17	6490	+	20	–
tetradecane	13.340	C_14_H_30_	85	57.11	0.07	12389	–	5	–
3-hexyne-2,5-diol, 2,5-dimethyl-	13.579	C_8_H_14_O_2_	172	109.1	0.07	8883	+	94	–
benzaldehyde, 2,4-dihydroxy-6-methyl-	13.794	C_8_H_8_O_3_	182	151.1	0.08	251690	+	96	+
1,2,7,8-octanetetrol	14.183	C_8_H_18_O_4_	97	55.08	0.05	103699	+	97	–
ethanone, 1-(3-hydroxy-4-methoxyphenyl)-	14.809	C_9_H_10_O_3_	136	151.1	0.03	95693	+	107	+
phenol, 3,5-bis(1,1-dimethylethyl)-	14.834	C_14_H_22_O	90	191.2	0.07	70825	+	108	+
(1*S*,2*S*,4*S*)-trihydroxy-*p*-menthane	14.878	C_10_H_20_O_3_	96	71.08	0.03	5326310	+	109	–
butanoic acid	15.485	C_4_H_8_O_2_	35	60.06	0.02	264	+	0	–
phenol, 4-pentyl-	15.586	C_11_H_16_O	24	107.1	0.01	26975	+	114	+
4-vinylbenzoic acid	16.308	C_9_H_8_O_2_	30	148.1	0.01	14098	+	117	–
2-heptanone	16.981	C_7_H_14_O	87	58.08	0.04	8051	–	0	–
2-cyclohexen-1-one, 4-(3-hydroxybutyl)-3,5,5-trimethyl-	17.230	C_13_H_22_O_2_	29	95.09	0.01	118284	+	126	–
*n*-decanoic acid	17.741	C_10_H_20_O_2_	149	60.06	0.05	2969	+	0	–
3,5-di*tert*-butyl-4-hydroxybenzaldehyde	17.771	C_15_H_22_O_2_	47	219.2	0.01	73219	+	132	+
13-tetradece-11-yn-1-ol	17.977	C_14_H_24_O_2_	45	67.09	0.03	543337	+	135	–
salicylic acid, *tert*-butyl ester	18.214	C_11_H_14_O_3_	11	120.0	0.00	11424104	+	138	+
2-cyclohexen-1-one, 4-hydroxy-3,5,6-trimethyl-4-(3-oxo-1-butenyl)-	18.248	C_13_H_18_O_3_	277	124.1	0.08	5371378	+	140	–
benzene, 1,1′-[1,2-ethanediylbis(oxy)]bis-	18.286	C_14_H_14_O_2_	47	77.07	0.04	7713	–	17	+
hexanoic acid	18.729	C_6_H_12_O_2_	44	60.06	0.03	8892	+	0	–
3,7,11,15-tetramethyl-2-hexadecen-1-ol	18.842	C_20_H_40_O	59	82.11	0.06	5366244	+	23	–
2-pentadecanone	19.046	C_15_H_30_O	97	58.09	0.06	61303	–	0	–
*n*-hexadecanoic acid	19.578	C_16_H_32_O_2_	3776	73.07	7.57	985	+	0	–
nonanamide	19.674	C_9_H_19_NO	200	59.08	0.23	70709	–	0	–
dodecane, 1-iodo-	20.130	C_12_H_25_I	57	57.11	0.31	20282	–	5	–
4,4′-(1,2-diethylethylene)diphenol	20.205	C_18_H_22_O_2_	35	135.2	0.02	192197	+	152	+
nonanoic acid	20.384	C_9_H_18_O_2_	87	73.07	0.12	8158	+	0	–
1-undecanol	20.522	C_11_H_24_O	12	69.11	0.05	8184	+	5	–
octa-3,5-diene-2,7-dione, 4,5-dihydroxy-	20.660	C_8_H_10_O_4_	35	85.06	0.06	5367917	+	154	–
phytol	20.747	C_20_H_40_O	236	71.09	0.44	5280435	+	23	–
dimethoxymethyl-hydroxy-triphenyl phosphide	20.924	C_21_H_23_O_3_P	49	277.2	0.03	624086	+	155	–
9,12-octadecadienoic acid (*Z*,*Z*)-	21.084	C_18_H_32_O_2_	665	67.1	4.20	5280450	+	0	–
*trans*-13-octadecenoic acid	21.124	C_18_H_34_O_2_	1056	55.1	16.0	6161490	+	0	–
oleic acid	21.316	C_18_H_34_O_2_	458	55.1	0.93	445639	+	0	–
nonanamide	21.348	C_9_H_19_NO	274	59.08	0.41	70709	–	0	–
nonanamide	21.514	C_9_H_19_NO	814	59.08	1.06	70709	–	0	–
citronellol epoxide (R or S)	22.325	C_10_H_20_O_2_	89	59.08	0.10	98467	+	162	–
decane, 1-iodo-	22.467	C_10_H_21_I	65	57.11	0.60	16314	–	5	–
3,4-hexanedione, 2,2,5,5-tetramethyl-, monoxime	22.707	C_10_H_19_NO_2_	67	57.11	0.38	9601652	+	164	–
nonane, 1-iodo-	23.428	C_9_H_19_I	23	57.12	0.97	20275	–	5	–
9-octadecenamide, (*Z*)-	23.796	C_8_H_35_NO	1360	59.08	4.67	5283387	–	0	–
13-docosenamide, (*Z*)-	23.881	C_22_H_43_NO	115	59.08	0.66	5365371	–	0	–

a (+) = Present (compound matches SMARTS rule or belongs to antioxidant cluster); (−) = Absent (no SMARTS match and not part of antioxidant cluster).

SMARTS-based screening identified a chemically diverse antioxidant subset dominated by phenolic derivatives, benzylic alcohols, oxygenated monoterpenoids, hydroxy-aromatic aldehydes, and medium-chain fatty acids. Phenolic compounds such as phenol, 4-ethyl- and phenol, 4-ethyl-2-methoxy- represent classical H-donor antioxidants, with methoxy- and alkyl-substitution enhancing resonance stabilization and radical-quenching efficiency.^47,48^ Sterically hindered phenols, including 3,5-bis­(1,1-dimethylethyl)­phenol and 3,5-di-*tert*-butyl-4-hydroxybenzaldehyde, resemble butylated hydroxytoluene (BHT)-type lipid antioxidants widely reported for strong peroxyl-radical inhibition.^49^

Benzylic and aromatic alcohols (benzyl alcohol, phenylethyl alcohol, 2-methoxybenzyl alcohol) form a secondary group consistent with moderate radical-scavenging activity frequently documented in essential oils.^50^ The data set also contained oxygenated monoterpenoids such as terpinen-4-ol and 1,4-dihydroxy-*p*-menth-2-ene, compounds noted for ROS-scavenging, anti-inflammatory, and membrane-protective effects.^51^ Hydroxy-aromatic aldehydes, including 2,4-dihydroxy-6-methylbenzaldehyde, exhibit catechol-like redox behavior, while medium-chain fatty acids contribute indirectly through modulation of oxidative pathways.

The structural clustering analysis grouped molecules sharing aromatic oxygenation and benzylic or methoxy-substituted motifs, even when SMARTS rules did not explicitly match. Clusters enriched with phenols, methoxy-phenols, benzyl alcohols, and dihydroxy aromatics aligned with well-established antioxidant pharmacophores.^52^ Representative examples include phenol, 4-ethyl- (cluster 55), 2-methoxybenzyl alcohol (cluster 70), and 2,4-dihydroxy-6-methylbenzaldehyde (cluster 96), confirming that similarity-based clustering successfully captures structural families associated with ROS-modulating activity.

Compounds detected by both approaches represent the most reliable antioxidant motifs in the extract. These included Phenol, 4-ethyl-, Ethanol, 2-phenoxy-, 2-methoxybenzyl alcohol, Phenol, 4-ethyl-2-methoxy-, 2,4-dihydroxy-6-methylbenzaldehyde, 1-(3-hydroxy-4-methoxyphenyl)­ethanone, 3,5-bis­(1,1-dimethylethyl)­phenol, Phenol, 4-pentyl- 3,5-di-*tert*-butyl-4-hydroxybenzaldehyde, 4,4′-(1,2-diethylethylene)­diphenol, and Salicylic acid tert-butyl ester. These molecules feature canonical antioxidant pharmacophores, which include phenolic OH groups, methoxy substituents, benzylic alcohols, and catechol/vanillyl-like frameworks and consistently appear within structurally coherent antioxidant clusters. Their dual identification reflects the presence of robust antioxidant chemotypes with well-documented radical scavenging, lipid-peroxidation inhibition, and electron-transfer properties.^15^ These molecules likely constitute the core antioxidant chemical space of the GC–MS extract.

#### 3.4.1 Antioxidant Scaffold Analysis

Scaffold analysis was performed to determine the dominant structural frameworks associated with antioxidant activity within the 66-compound data set. Bemis–Murcko scaffolds were generated for all molecules, allowing the reduction of each structure to its core ring and linker system. Scaffold frequency analysis revealed distinct chemical motifs that recur among antioxidant-associated compounds. Scaffold analysis of the antioxidant data set revealed clear structural dominance patterns (Figure 7). The top scaffold appeared 15 times, representing a major aromatic ring system broadly associated with radical-scavenging potential in natural products and essential oils.^53^ Secondary scaffolds occurred with lower frequencies (Scaffold 2:3; Scaffold 3:2; Scaffold 4:2; others: 1 occurrence each), indicating a chemically diverse set of antioxidant candidates within the GC–MS profile. While certain scaffolds were more frequent among the GC–MS-identified compounds, scaffold frequency alone does not directly indicate bioactivity. The potential of these scaffolds complements the molecular docking simulation and experimental assays, allowing a more informed evaluation of scaffold–activity relationships.

**Figure 7:**
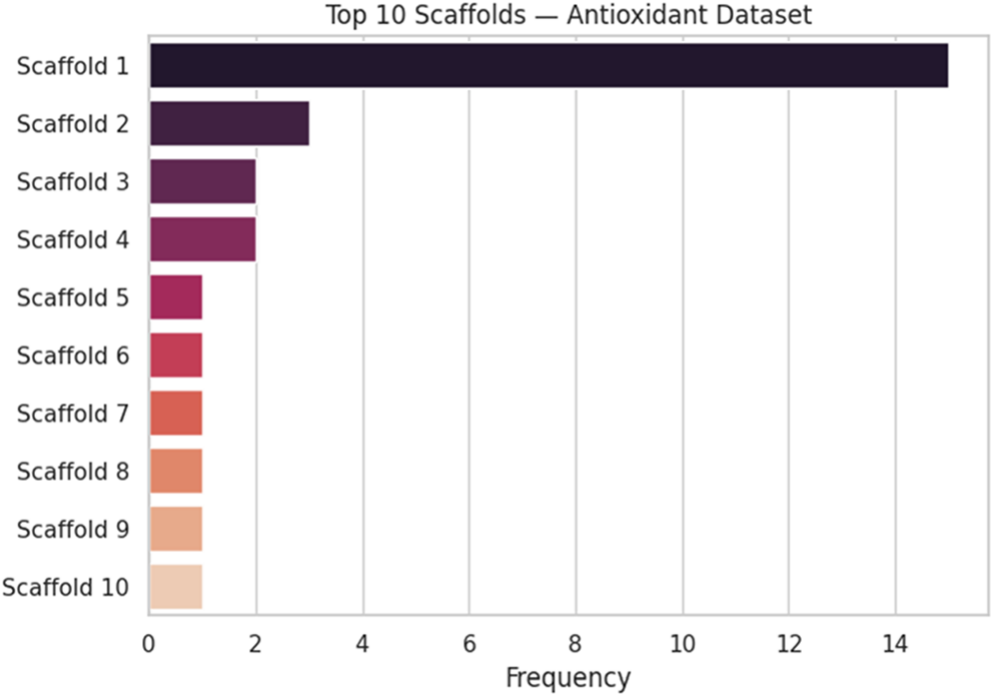
Distribution of the top 10 scaffolds in the antioxidant data set.

Figure 8 represents ten distinct core structural frameworks identified in the antioxidant-associated GC–MS data set of *C. siliqua* extract. The most abundant motif was the aromatic benzene scaffold, underscoring the dominance of phenyl systems whose π-electron delocalization confers well-established radical-stabilizing properties. Saturated and unsaturated cyclohexyl scaffolds constituted the second and third most frequent classes, respectively, consistent with monoterpenoid backbones commonly implicated in plant-derived antioxidant activity. An oxygenated cyclohexanone scaffold also emerged among the dominant motifs, indicating the presence of α,β unsaturated carbonyl systems capable of engaging in electron-transfer-driven redox processes. The detection of a dioxolane heterocycle further highlighted the contribution of compact, oxygen-rich ring systems to antioxidant behavior. Additional low-frequency scaffolds included bicyclic terpenoid frameworks, polyfunctional cyclohexyl motifs, an indole-based heteroaromatic core, and a diphenyl ether. Collectively, these scaffolds demonstrate that the antioxidant potential of the extract is distributed across aromatic resonance systems, hydroxylated terpenoids, conjugated carbonyl functionalities, bicyclic carbocycles, and heteroatom-enriched rings, reflecting the structural heterogeneity characteristic of bioactive phytochemical mixtures.

**Figure 8:**
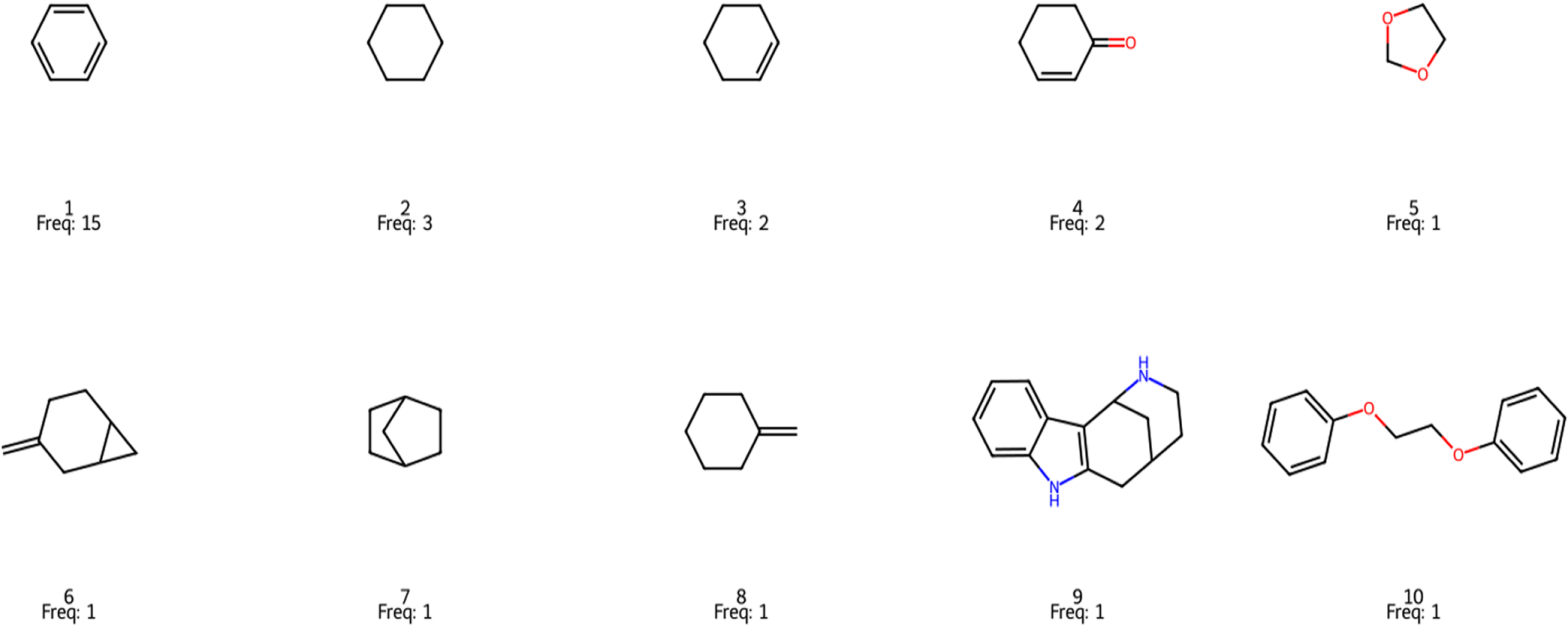
Ten distinct scaffolds identified in the antioxidant-associated GC–MS data set of *C. siliqua* L. extract.

To illustrate the chemical drivers represented by these structural frameworks, one representative compound was selected from each of the ten scaffolds shown in Figure 8. From the dominant aromatic phenyl scaffold (Scaffold 1), phenol, 4-ethyl- was identified as the key representative. The saturated cyclohexyl framework (Scaffold 2) was exemplified by cyclohexanol, 1-ethenyl, while the unsaturated cyclohexenyl scaffold (Scaffold 3) was represented by terpinen-4-ol. The oxygenated cyclohexanone class (Scaffold 4) was characterized by 2-cyclohexen-1-one, 4-(3-hydroxybutyl)-3,5,5-trimethyl-, and the heterocyclic dioxolane motif (Scaffold 5) by 1,3-dioxolane-2-methanol. The bicyclic terpenoid scaffold (Scaffold 6) was represented by *trans*-3­(10)-caren-2-ol, whereas the norbornane-type carbocycle (Scaffold 7) corresponded to bicyclo[2.2.1]­heptan-2-ol, 6-*tert*-butyl-. A polyfunctional cyclohexyl structure (Scaffold 8) was illustrated by 2-methyl-5-(propan-2-ylidene)­cyclohexane-1,4-diol, while the heteroaromatic indole-based scaffold (Scaffold 9) was represented by dasycarpidol. Finally, the diphenyl ether-like aromatic system (Scaffold 10) was exemplified by benzene, 1,1′-[1,2-ethanediylbis­(oxy)]­bis. Together, these ten compounds capture the structural breadth underlying the antioxidant potential inferred from the GC–MS scaffold distribution (Figure 9).

**Figure 9:**
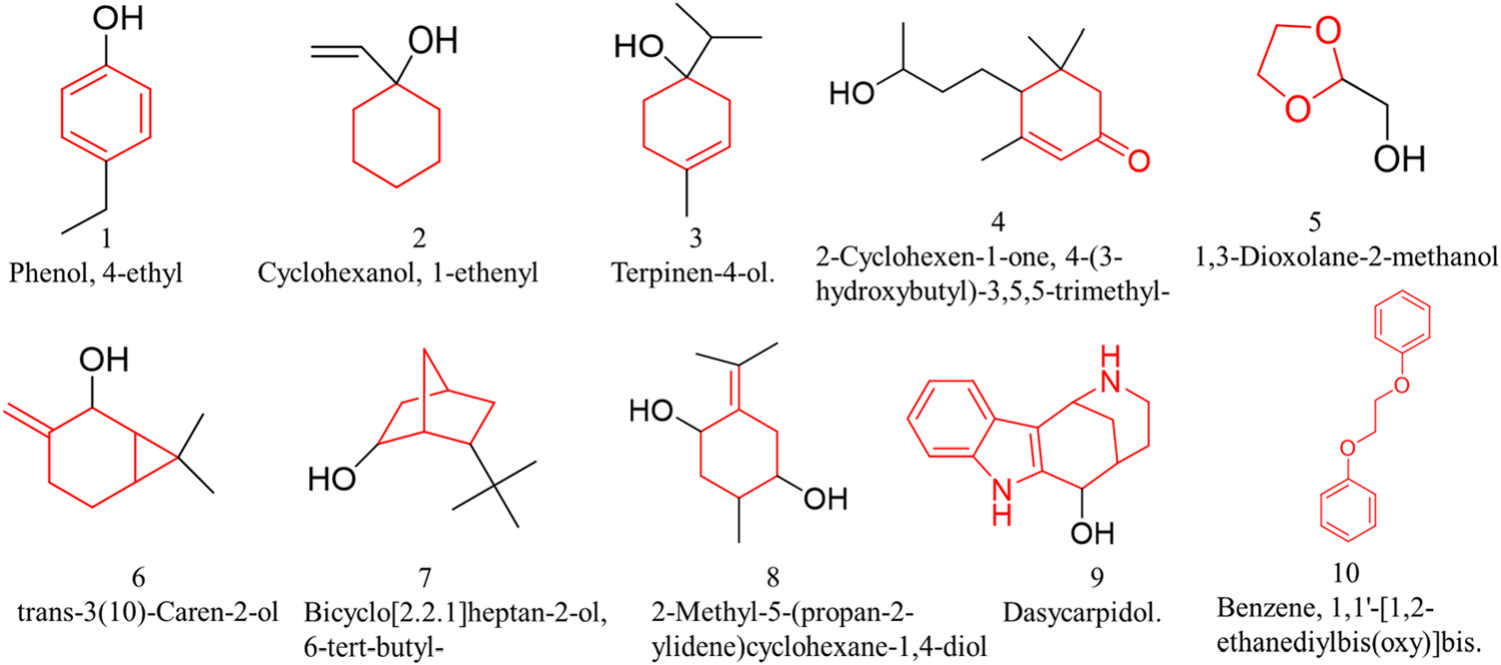
Chemical structures of the top 10 representative antioxidant-associated compounds, each annotated with its corresponding identified scaffold highlighted in red. Structures were generated using ChemDraw.

#### 3.4.2 Molecular Docking Studies of Antioxidant-Associated Compounds

Molecular docking was performed using Kelch-like ECH-associated protein 1 (KEAP1) (PDB ID: 5WG1), a key regulator of the antioxidant response through its interaction with Nrf2. Disruption of the KEAP1–Nrf2 interaction promotes activation of antioxidant defense pathways, making KEAP1 a relevant target for evaluating antioxidant-associated compounds (Figure 9).^38^

Table 2 shows the binding energies and inhibition constants derived from molecular docking studies of the selected GC–MS compounds with the identified antioxidant scaffold (Figure 9), and ascorbic acid as a reference compound when complexed with KEAP1. The selected compounds exhibited binding affinities ranging from −5.3 to −6.8 kcal/mol. Among them, dasycarpidol and benzene, 1,1′-[1,2-ethanediylbis­(oxy)]­bis appear to be the most potent compounds against KEAP1, with a binding energy of −6.8 kcal/mol (*K*_*i*_ of 10.382 μM) and −6.6 kcal/mol (*K*_*i*_ of 14.463 μM), respectively, outperforming the reference compound, ascorbic acid (−5.7 kcal/mol). 2-Methyl-5-(propan-2-ylidene)­cyclohexane-1,4-diol and 2-cyclohexen-1-one, 4-(3-hydroxybutyl)-3,5,5-trimethyl- followed with binding energies of −5.9 kcal/mol (*K*_*i*_ = 48.020 μM) and −5.8 kcal/mol (*K*_*i*_ = 57.331 μM), respectively, while phenol, 4-ethyl-, terpinen-4-ol, and *trans*-3­(10)-caren-2-ol displayed binding within a range similar to the reference compound, suggesting their potential contribution to the overall antioxidant activity of the extract. The observed binding patterns suggest that selected phytochemicals may contribute to antioxidant effects through possible modulation of the KEAP1–Nrf2 pathway, supporting the bioactivity findings of the *C. siliqua* pod extract.

**Table 2:** Molecular Docking Results for the Top Antioxidant-Associated Compounds and Reference Standard Drugs against KEAP1 Target

ligand	binding energy (kcal/mol) (5WG1)	inhibition constant (μM)
phenol, 4-ethyl-	–5.6	79.042
terpinen-4-ol	–5.3	130.181
*trans*-3(10)-caren-2-ol	–5.5	94.093
2-cyclohexen-1-one, 4-(3-hydroxybutyl)-3,5,5-trimethyl-	–5.8	57.331
ascorbic acid	–5.7	67.335
2-methyl-5-(propan-2-ylidene)cyclohexane-1,4-diol	–5.9	48.020
dasycarpidol	–6.8	10.382
benzene, 1,1′-[1,2-ethanediylbis(oxy)]bis	–6.6	14.463

Figure 10 illustrates the molecular interactions of the top-performing compound (highest binding affinity) alongside the two compounds with binding energies comparable to ascorbic acid in complex with KEAP1 (5WG1). All studied compounds exhibited π–alkyl interactions with Tyr334, along with multiple hydrogen bonds within the protein’s binding pocket, indicating its stability (Figure 10). Hydrogen bonding is a key determinant of strong protein–ligand interactions, and it enhances binding affinity and inhibitory potency. These interactions are supported by hydroxyl groups and conjugated systems, which are known to enhance molecular stability and interaction within protein binding pockets. Notably, the interactions are localized within the Nrf2 binding groove, a critical hotspot on KEAP1, involving key residues such as Arg415, Ser508, Ser363, and Arg380 ^38^ The positioning of these ligands within this region suggests their potential to interfere with the KEAP1–Nrf2 interaction, thereby promoting the activation of antioxidant defense pathways.

**Figure 10:**
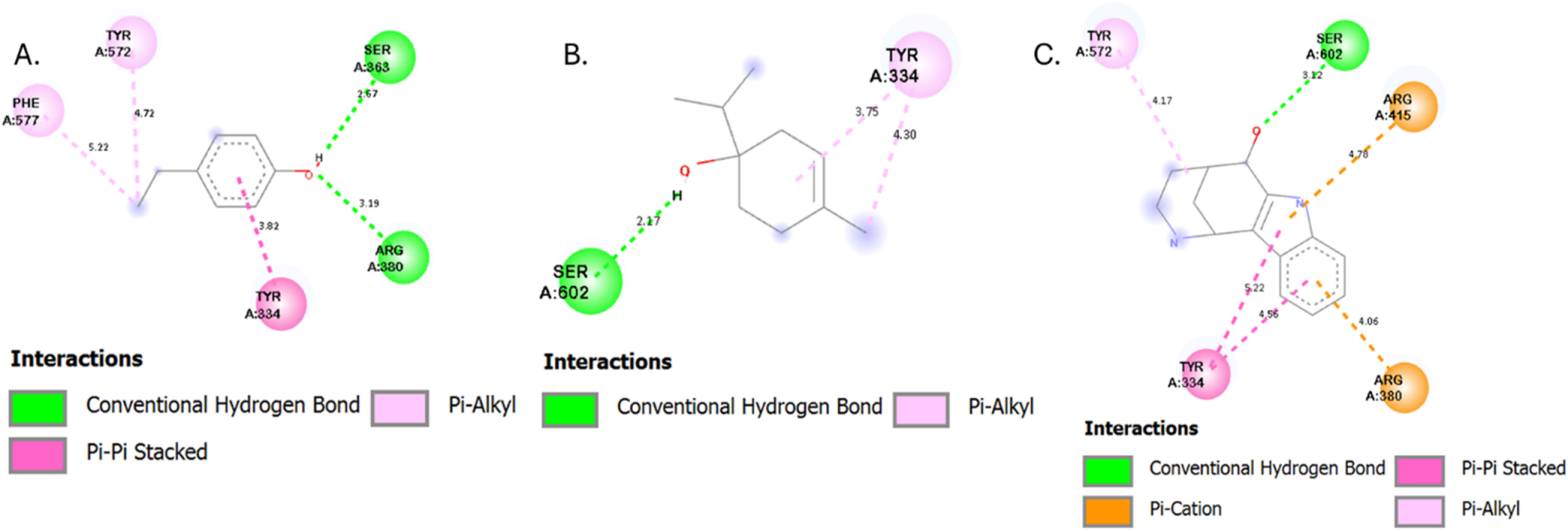
2D interaction of (A) phenol, 4-ethyl-, (B) terpinen-4-ol, and (C) dasycarpidol with Kelch-like ECH-associated protein 1 (5WG1).

#### 3.4.3 Antioxidant Activity of *C. siliqua* L. Pod Extract

The antioxidant capacity of the *Ceratonia siliqua* L. (*C. siliqua*) hexane extract was evaluated using the DPPH free-radical-scavenging assay. The extract showed clear radical-quenching activity across the tested concentrations, confirming the presence of antioxidant constituents. The calculated IC_50_ value for the extract was 12.81 ± 1.08 μg/mL, whereas ascorbic acid, which was used as the positive control, displayed a much stronger response with an IC_50_ of 1.968 ± 0.02 μg/mL. This difference was statistically significant (*p* < 0.001, one-way ANOVA, Tukey test).

Although less potent than ascorbic acid, the *C. siliqua* extract still showed meaningful free-radical-scavenging activity, indicating that the hexane fraction contains compounds capable of donating hydrogen atoms or electrons to neutralize DPPH radicals. These findings align with the phytochemical profile of *C. siliqua*, which is known to contain terpenoids and phenolic-related volatiles that may contribute to its antioxidant activity.^54,55^ As shown in Figure 11, the extract exhibits a gradual, dose-dependent increase in DPPH inhibition, reaching high activity only at the upper concentrations, whereas ascorbic acid rises sharply and achieves near-complete inhibition at much lower doses. The spacing between the curves reflects the IC_50_ values, confirming the greater potency of ascorbic acid while demonstrating that the *C. siliqua* extract still possesses a notable antioxidant capacity.

**Figure 11:**
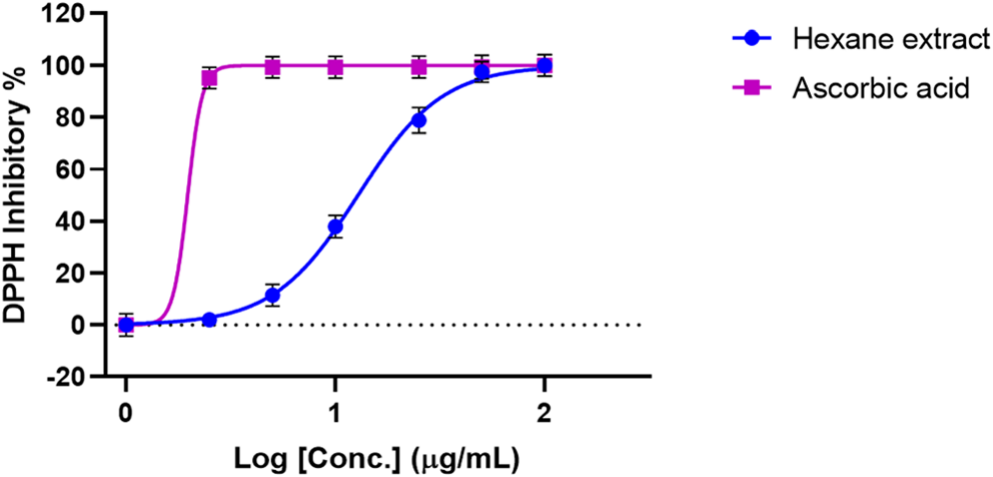
Dose-dependent DPPH scavenging curves for the *C. siliqua* hexane extract and ascorbic acid. IC_50_ values were 12.81 ± 1.08 μg/mL for *C. siliqua* and 1.968 ± 0.02 μg/mL for ascorbic acid (positive control). Values are expressed as mean ± SD (*n* = 3); at *p* < 0.001 using one-way ANOVA followed by Tukey test.

Therefore, the ten representative compounds (Figure 9) selected from the dominant scaffolds capture the chemical space most likely driving the DPPH antioxidant response. Although the extract is less potent than ascorbic acid, the phenolic- and terpenoid-rich nature of these scaffolds offers a clear mechanistic basis for the observed radical-scavenging activity. In particular, GC–MS-identified constituents from the identified antioxidant scaffolds, such as terpinen-4-ol has been widely reported to exhibit significant antioxidant activity by showing scavenging activity against 2,2-diphenyl-1-picrylhydrazyl (DPPH) free radicals, as well as modulating oxidative stress, which is associated with diseases including neurodegenerative disorders, cancers, cardiovascular diseases, diabetes, and inflammatory conditions.^56,57^ Similarly, phenolic compounds such as phenol, 4-ethyl- are well-known for their hydrogen-donating ability and antioxidant potential.^58,59^ In addition, plant monoterpenoid components such as *trans*-3­(10)-caren-2-ol and oxygenated terpenoid derivatives (2-cyclohexen-1-one, 4-(3-hydroxybutyl)-3,5,5-trimethyl-) have also been reported to contribute to antioxidant activity.^60^ These findings directly support the antioxidant effect measured in the assay.

### 3.5 GC–MS Antimicrobial Data Set

GC–MS profiling of *C. siliqua* L. revealed a diverse set of volatile constituents associated with antimicrobial activity. SMARTS-based substructure screening identified 29 phytochemicals containing structural motifs commonly linked to antibacterial and antifungal effects (Table 3). Subsequent structural clustering using ECFP4 fingerprints (Tanimoto >0.6) confirmed that all of these SMARTS-matched compounds already belonged to well-defined and biologically meaningful chemotypes. In other words, the clustering step did not introduce any additional molecules, demonstrating that the SMARTS rules successfully captured the entire antimicrobial chemical space present in the extract.

**Table 3:** GC–MS-Identified Antimicrobial Compounds Based on SMARTS Pharmacophores and Structural Clustering^a^

name	R.T. (min)	formula	peak S/N	base mass	area (%)	PubChem_CID	antimicrobial (SMART)	Cluster ID	antimicrobial (cluster)
d-limonene	7.699	C_10_H_16_	408	68.1	0.28	440917	+	29	–
*a*-phellandrene	8.192	C_10_H_16_	125	93.1	0.11	7460	+	33	–
dihydro-3-methylene-5-methyl-2-furanone	8.596	C_6_H_8_O_2_	1411	68.06	0.71	99939	+	35	–
bicyclo[2.2.1]heptane, 7,7-dimethyl-2-methylene-	8.978	C_10_H_16_	23	93.1	0.02	28930	+	41	–
benzene, (methylenecyclopropyl)-	9.840	C_10_H_10_	14	128.09	0.01	141510	+	48	–
dehydromevalonic lactone	10.114	C_6_H_8_O_2_	471	82.07	0.37	557445	+	52	–
terpinen-4-ol	10.282	C_10_H_18_O	1617	71.09	1.29	11230	+	53	–
1*H*-pyrrole-2,5-dione, 3-ethyl-4-methyl-	11.36	C_7_H_9_NO_2_	41	67.1	0.02	29995	+	66	+
2,6-octadiene-4,5-diol, 3,6-dimethyl-	11.37	C_10_H_18_O_2_	321	85.06	0.12	5362856	+	67	–
2-methylbenzoic acid, 2,4-dichloronaphthyl-1 ester	11.73	C_18_H_12_Cl_2_O_2_	27	119.11	0.02	91710400	+	71	+
*trans*-3(10)-caren-2-ol	12.149	C_10_H_16_O	249	71.09	0.16	572861	+	78	–
2-methyl-5-(propan-2-ylidene)cyclohexane-1,4-diol	12.368	C_10_H_18_O_2_	393	74.07	0.19	91697160	+	81	–
2-butenal, 2-methyl-	12.650	C_5_H_8_O_2_	33	84.05	0.03	5321950	+	85	–
4,6,6-trimethyl-5,6-dihydro-2*H*-pyran-2-one	13.504	C_8_H_12_O_2_	182	82.07	0.05	247461	+	93	–
2-methyl-6-methyleneoct-7-en-3-one	14.467	C_10_H_16_O	74	71.09	0.06	85845089	+	102	–
2(4*H*)-benzofuranone, 5,6,7,7a-tetrahydro-4,4,7a-trimethyl-, (R)-	15.249	C_11_H_16_O_2_	118	111.07	0.04	6432173	+	111	–
nonane, 2,8-dimethyl-4-methylene-	16.754	C_12_H_24_	21	83.12	0.01	544668	+	123	–
cyclohexene, 1,5,5-trimethyl-6-(2-propenylidene)-	16.840	C_12_H_18_	39	45.07	0.04	5372935	+	124	–
2,3-dihydro-1,3-dimethyl(1*H*)cyclopenta[*b*]quinoxaline	16.868	C_13_H_14_N_2_	32	183.17	0.01	603342	+	125	–
2-cyclohexen-1-one, 4-(3-hydroxybutyl)-3,5,5-trimethyl-	17.230	C_13_H_22_O_2_	29	95.09	0.01	118284	+	126	–
2-cyclohexen-1-one, 4-hydroxy-3,5,6-trimethyl-4-(3-oxo-1-butenyl)-	18.248	C_13_H_18_O_3_	277	124.09	0.08	5371378	+	140	–
neophytadiene	18.449	C_20_H_38_	130	68.1	0.07	10446	+	142	–
3,7,11,15-tetramethyl-2-hexadecen-1-ol	18.842	C_20_H_40_O	59	82.11	0.06	5366244	+	23	–
(*E*,*E*)-7,11,15-trimethyl-3-methylene-hexadeca-1,6,10,14-tetraene	19.146	C_20_H_32_	139	69.11	0.17	5365895	+	147	–
7,9-di*tert*-butyl-1-oxaspiro(4,5)deca-6,9-diene-2,8-dione	19.170	C_17_H_24_O_3_	68	57.11	0.09	545303	+	148	–
2,4,6-tris(1,1-dimethylethyl)-4-methylcyclohexa-2,5-dien-1-one	20.051	C_19_H_32_O	142	205.14	0.04	609653	+	151	–
phytol	20.747	C_20_H_40_O	236	71.09	0.44	5280435	+	23	–
2,2,4,5-tetramethyl-5-hexen-3-one	21.940	C_10_H_18_O	29	57.11	0.09	11815964	+	160	–
bicyclo[2.2.1]hept-2-ene, 2,7,7-trimethyl-	22.977	C_10_H_16_	57	93.1	0.13	564720	+	166	–

a (+) = Present (compound matches SMARTS rule or belongs to antimicrobial cluster); (−) = Absent (no SMARTS match and not part of antimicrobial cluster).

The antimicrobial profile of *C. siliqua* was dominated by monoterpenoids such as d-limonene, terpinen-4-ol, and *trans*-3­(10)-caren-2-ol, together with several bicyclic terpenes, including bicyclo[2.2.1]­heptane derivatives. These hydrophobic terpenes are well-known for their ability to integrate into microbial membranes, increasing permeability, and disrupting quorum-regulated behaviors, a mechanism widely documented for monoterpenes in essential oils.^61–63^

In addition to these terpene-based constituents, the extract contained oxygenated small molecules such as dihydro-3-methylene-5-methyl-2-furanone, dehydromevalonic lactone, 2*H*-pyran-2-one derivatives, electrophilic enones, and short-chain diols.^64,65^ These scaffolds commonly introduce redox-active or electrophilic functionalities that can interfere with microbial metabolism through thiol reactivity, oxidative stress, or enzyme inhibition.^66^

More structurally complex metabolites, including benzofuranones, substituted aromatics, pyrrolidinediones, neophytadiene, and phytol, further enriched the antimicrobial chemical space. Such scaffolds have been associated with lipid–protein interactions, metabolic enzyme inhibition, and modulation of oxidative pathways in various microbial systems.^67–69^

Overall, the antimicrobial landscape of *C. siliqua* reflects an interplay between membrane-active terpenes and oxygenated or heterocyclic metabolites capable of perturbing microbial metabolism or redox balance. This diversity of mechanisms underscores the biological relevance of the identified compounds and strengthens their potential value for the downstream antimicrobial prioritization.

#### 3.5.1 Antimicrobial Scaffold Analysis

Scaffold analysis of the 29 antimicrobial compounds revealed a small number of recurring structural frameworks (Figure 12). The top three scaffolds (Scaffolds 1–3) each appeared twice and corresponded mainly to monoterpenoid, and bicyclic terpene cores commonly associated with antimicrobial activity in plant–derived metabolites. The remaining scaffolds (Scaffolds 4–10) occurred once each, reflecting additional but less frequent contributions from oxygenated terpenoids, heterocycles, and aromatic derivatives. Overall, the antimicrobial data set is dominated by a few terpene-based scaffolds, with the remaining structures contributing single, unique frameworks that broaden the chemical diversity of the antimicrobial space.

**Figure 12:**
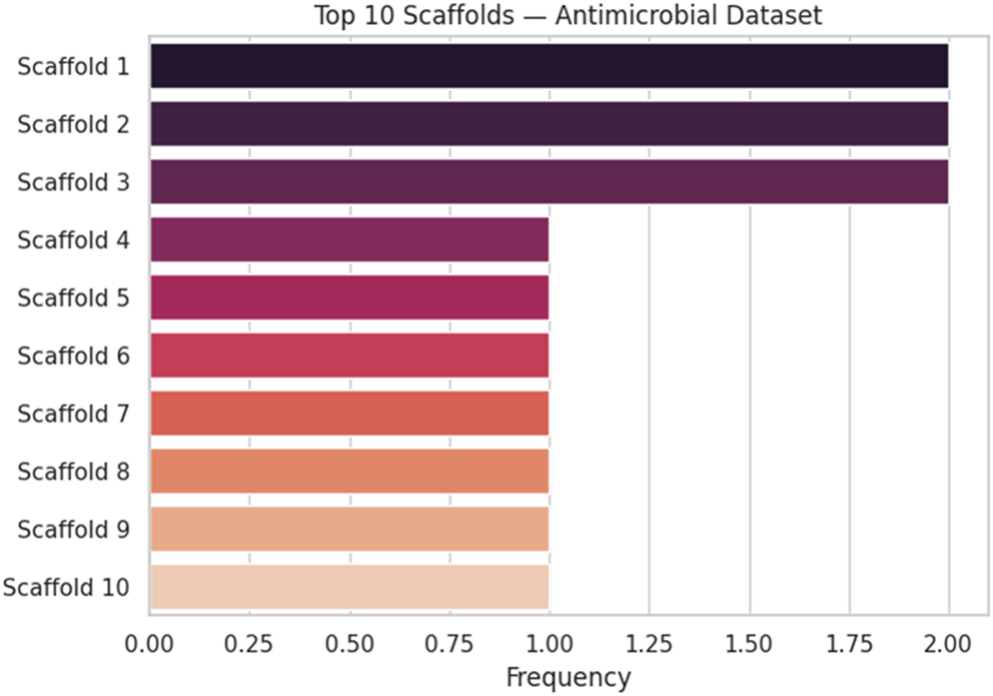
Distribution of the top 10 scaffolds in the antimicrobial data set.

Figure 13 presents the ten Bemis–Murcko scaffolds identified within the antimicrobial subset of the *C. siliqua* GC–MS profile. The most frequent frameworks (Scaffolds 1–4) correspond to simple cyclohexyl and oxygenated cyclohexanone cores, consistent with monoterpenoid backbones commonly associated with antimicrobial activity in plant volatiles.^70^ The remaining scaffolds occurred once each and included small bicyclic terpenes, fused-ring carbocycles, lactone-like motifs, and an aromatic ester scaffold. These structures reflect the broader diversity of antimicrobial chemotypes present in the extract, spanning nonoxygenated terpene rings, mildly electrophilic oxygenated cyclic systems, and more rigid aromatic or bicyclic frameworks. Overall, the scaffold distribution highlights a terpene-dominant antimicrobial space complemented by a limited number of oxygenated and aromatic cores, representing a compact yet structurally varied set of antimicrobial scaffolds.

**Figure 13:**
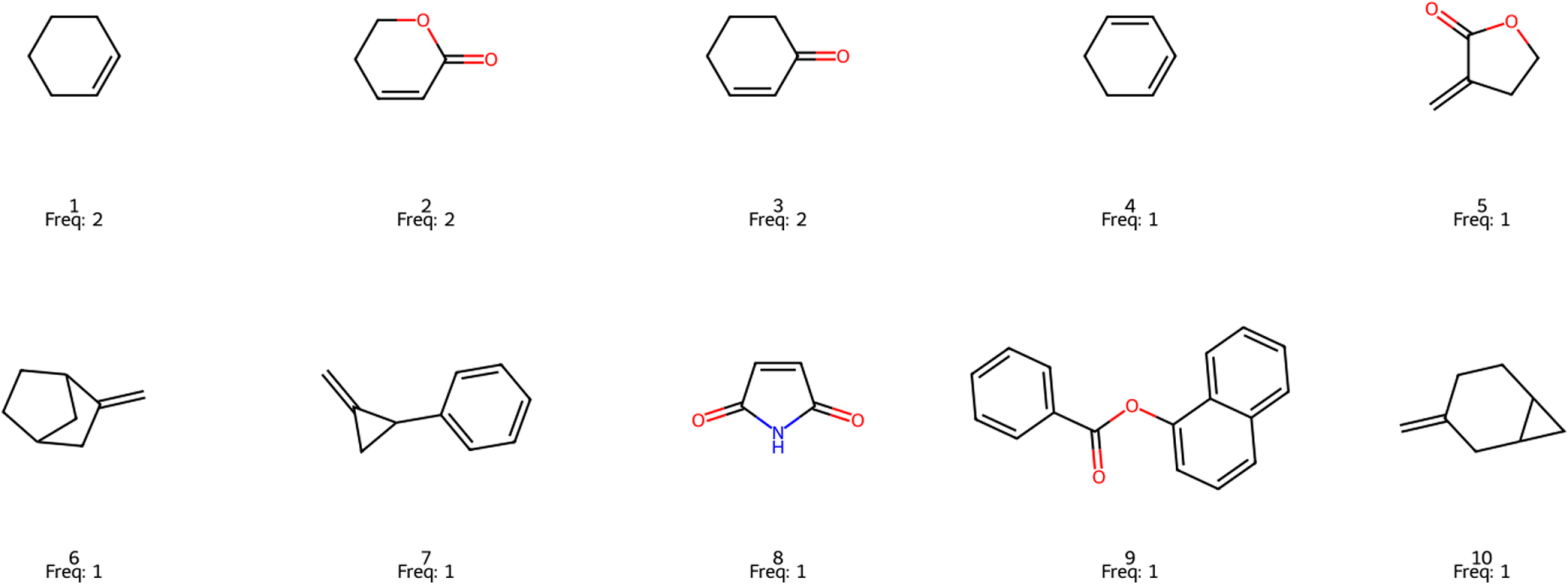
Ten distinct scaffolds identified in the antimicrobial-associated GC–MS data set of *C. siliqua* L. extract.

To show the chemical features captured by the ten antimicrobial scaffolds, one representative compound was selected for each framework in Figure 13. The dominant monoterpene scaffold (Scaffold 1) was represented by d-limonene and terpinen-4-ol, both widely reported antimicrobial terpenoids.^71,72^ The oxygenated lactone 4,6,6-trimethyl-5,6-dihydro-2H-pyran-2-one illustrated Scaffold 2, while 2-cyclohexen-1-one, 4-(3-hydroxybutyl)-3,5,5-trimethyl- represented the α,β-unsaturated cyclohexanone framework of Scaffold 3.

Scaffold 4 was exemplified by the monoterpene α-phellandrene,^73^ and Scaffold 5 by the small heterocycle dihydro-3-methylene-5-methyl-2-furanone. The strained aromatic–cyclopropyl system benzene, (methylenecyclopropyl)- represented Scaffold 7, while Scaffold 8 corresponded to 1*H*-pyrrole-2,5-dione, 3-ethyl-4-methyl-. For Scaffold 9, the representative compound was the aromatic ester 2-methylbenzoic acid, 2,4-dichloronaphthyl ester. The bicyclic monoterpenol trans-3(10)-caren-2-ol illustrated Scaffold 10. These representative structures (Figure 14) reflect the mixture of monoterpenes, oxygenated rings, and small heterocycles that define the antimicrobial chemical space of *C. siliqua*.

**Figure 14:**
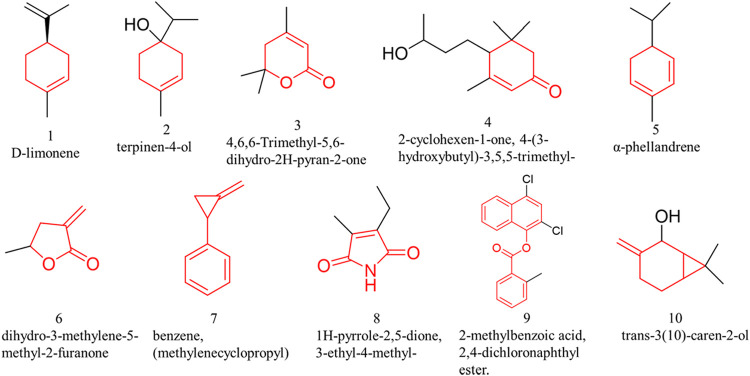
Chemical structures of the top 10 representative antimicrobial-associated compounds, each annotated with its corresponding identified scaffold highlighted in red. Structures were generated using ChemDraw.

#### 3.5.2 Molecular Docking Studies of Antimicrobial-Associated Compounds

Molecular docking was performed to evaluate the interaction of antimicrobial-associated compounds (Figure 14) with *S. aureus* dihydrofolate reductase (DHFR) (PDB ID: 3FRD), a key enzyme responsible for the increase in affinity and antibacterial activity. Inhibition of DHFR disrupts nucleotide biosynthesis, thereby impairing DNA replication and microbial proliferation, making it a relevant target for antimicrobial drug discovery.^74^ The docking results (Table 4) reveal that the reference compound, chlorhexidine (−9.8 kcal/mol, *K*_*i*_ = 0.0640 μM), showed higher affinity than gentamicin (−7.9 kcal/mol, *K*_*i*_ = 1.6370 μM). Notably, the identified antimicrobial-associated compounds (2-methylbenzoic acid, 2,4-dichloronaphthyl ester) exhibited the strongest binding affinity (−9.9 kcal/mol, *K*_*i*_ = 0.0550 μM), slightly outperforming both reference compounds, followed by other ligands from the identified antimicrobial scaffolds, including 2-cyclohexen-1-one, 4-(3-hydroxybutyl)-3,5,5-trimethyl- (−6.7 kcal/mol, *K*_*i*_ = 12.288 μM), trans-3(10)-caren-2-ol (−6.4 kcal/mol, *K*_*i*_ = 20.254 μM), and benzene (methylenecyclopropyl)- (−5.9 kcal/mol, *K*_*i*_ = 48.020 μM).

**Table 4:** Molecular Docking Results for the Top Antimicrobial-Associated Compounds and Reference Standard Drugs against Staphylococcus aureus DHFR Target

ligand	binding energy (kcal/mol) (3FRD)	inhibition constant (μM)
2-cyclohexen-1-one, 4-(3-hydroxybutyl)-3,5,5-trimethyl-	–6.7	12.288
terpinen-4-ol	–5.6	79.042
*trans*-3(10)-caren-2-ol	–6.4	20.254
2-methylbenzoic acid, 2,4-dichloronaphthyl ester	–9.9	0.0550
benzene, (methylenecyclopropyl)-	–5.9	48.020
α-phellandrene	–5.7	67.335
chlorhexidine	–9.8	0.0640
gentamicin	–7.9	1.6370

Figure 15 illustrates the molecular interactions of the top three antimicrobial-associated compounds and the reference drug with 3FRD. Binding mode analysis reveals key interactions within the active site, involving important residues such as Arg57, Leu54, Phe92, Ile50, Trp22, Asp27, and Val31, which are known to contribute to ligand stabilization within the DHFR binding pocket.^39^ 2-Cyclohexen-1-one, 4-(3-hydroxybutyl)-3,5,5-trimethyl- and 2-methylbenzoic acid, 2,4-dichloronaphthyl ester formed hydrogen bonds with Thr46 (2.98 Å and 2.94 Å, respectively), indicating stable interactions. The reference compound, chlorhexidine, exhibited the highest number of hydrogen bonds (3), while the top-performing compounds formed two hydrogen bonds each (Figure 15). In contrast, *trans*-3­(10)-caren-2-ol showed no hydrogen bonding but maintained hydrophobic interactions within the binding pocket. These findings suggest that certain phytochemicals such as 2-methylbenzoic acid, 2,4-dichloronaphthyl ester and 2-cyclohexen-1-one derivatives possess favorable binding interactions with DHFR and may contribute to the observed antimicrobial activity. However, despite strong binding by some individual compounds, the overall antimicrobial effect of the crude extract is likely influenced by factors such as compound concentration, bioavailability, and possible synergistic or antagonistic interactions.

**Figure 15:**
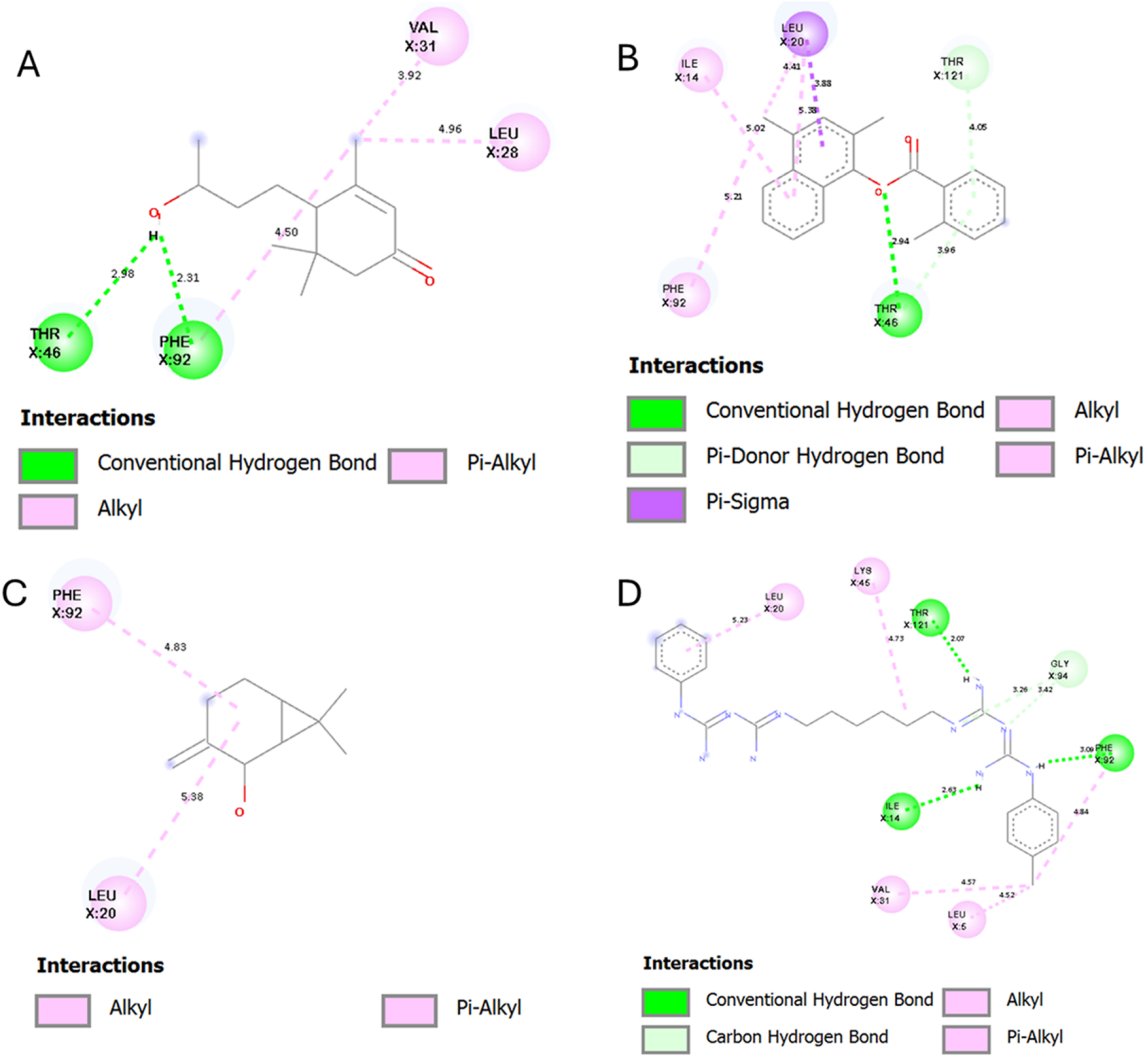
2D interaction of (A) 2-cyclohexen-1-one, 4-(3-hydroxybutyl)-3,5,5-trimethyl-, (B) 2-methylbenzoic acid, 2,4-dichloronaphthyl ester, (C) *trans*-3­(10)-caren-2-ol, and the standard drug (D) chlorhexidine with *S. aureus* DHFR (3FRD).

#### 3.5.3 Antimicrobial Activity of *C. siliqua* L. Pod Extract

The inhibitory efficacy of the *C. siliqua* extract against the microorganism expressed moderate to poor results (Table 5). Moderate activity was observed against *S. aureus* with an MIC value of 625 μg/mL. In contrast, poor activity was noted against *E. coli* and *C. albicans*, which had MIC values of 2500 μg/mL and 5000 μg/mL, respectively. As for the positive controls, gentamicin and chlorhexidine, gentamicin exhibited a significant decrease in microbial viability, with MIC values of 31.52 μg/mL and 15.62 μg/mL against *S. aureus* and *E. coli*, respectively, whereas the chlorhexidine showed an MIC of 7.81 μg/mL for *C. albicans*.

**Table 5:** Antimicrobial MIC Values of C. siliqua Hexane Extracts against *S. aureus*, *E. coli*, and *C. albicans* ^a^

	MIC (μg/mL)		
extracts	*S. aureus*	*E. coli*	*C. albicans*
*C. siliqua* (Hex)	625	2500	5000
gentamicin	31.52	15.62	-
chlorhexidine	-	-	7.81

a Values are expressed as the mean of triplicate analyses (*n* = 3) ± standard deviation.

Previous research has indicated that hexane extracts can inhibit microorganisms by disrupting their membranes. This mechanism is attributed to nonpolar compounds, such as terpenoids and fatty acids, which penetrate the phospholipid bilayer of microbial cells.^75^ Additionally, terpenoids are well-known for their capacity to interfere with the H^+^-ATPase enzyme. This interference results in intracellular acidification and depletion of cellular components, contributing to the antimicrobial effects observed.^76^

Therefore, the top ten representative compounds selected from the dominant antimicrobial scaffolds (Figure 14) capture the chemical space most likely contributing to the inhibitory effects observed in the MIC assays. Notably, several GC–MS-identified compounds that map onto these scaffolds such as d-limonene and terpinen-4-ol have been widely reported to exhibit significant antibacterial and antibiofilm activity against *S. aureus*.^77,78^ Similarly, α-phellandrene is also known to contribute to antimicrobial effects against *Penicillium cyclopium* by altering the morphology of *P. cyclopium* hyphae, thereby causing loss of cytoplasmic material and distortion of the mycelia.^79^ These constituents’ frameworks dominate the antimicrobial scaffold space. These structures, along with the oxygenated lactones and small heterocycles represented within the scaffold set, align with the moderate inhibitory response detected against *S. aureus*, directly supporting the antimicrobial effect observed in the assay.

### 3.6 GC–MS Cytotoxic Data Set

GC–MS profiling of *C. siliqua* L. revealed a diverse set of volatile constituents associated with cytotoxic activity. SMARTS pattern screening identified 14 cytotoxic-related compounds in the GC–MS data set (Table 6). To include structurally similar molecules, cluster expansion using ECFP4 fingerprints (Tanimoto >0.6) added 14 more, giving a final set of 28 cytotoxic compounds. These molecules represent a small but structurally diverse group compared to the antioxidant and antimicrobial classes.

**Table 6:** GC–MS-Identified Cytotoxic Compounds Based on SMARTS Pharmacophores and Structural Clustering^a^

name	R.T. (min)	formula	peak S/N	base mass	area (%)	PubChem_CID	cytotoxic (SMART)	Cluster ID	cytotoxic (cluster)
butanoic acid	7.032	C_4_H_8_O_2_	155	60.06	0.09	264	–	0	+
1,2-hydrazinedicarboxaldehyde	7.056	C_2_H_4_N_2_O_2_	36	60.06	0.09	12342	+	26	–
hexanoic acid	7.236	C_6_H_12_O_2_	3442	60.06	7.14	8892	–	0	+
2-nonanone	8.714	C_9_H_18_O	246	58.08	0.13	13187	–	0	+
2-azetidinone, 1-*tert*-butyl-3,3-dimethyl-4-phenyl-	8.761	C_15_H_21_NO	24	117.1	0.02	583385	+	36	–
pyrrolidine-1-acetonitrile, 2,5-dioxo-	9.125	C_6_H_6_N_2_O_2_	56	56.1	0.02	544954	+	43	+
succinimide	9.679	C_4_H_5_NO_2_	67	56.1	0.02	11439	+	47	–
2-heptanone	10.35	C_7_H_14_O_2_	56	58.08	0.03	8051	–	0	+
2-oxetanone, 4-methylene-	10.81	C_4_H_4_O_2_	34	56.08	0.03	12661	+	58	–
1*H*-pyrrole-2,5-dione, 3-ethyl-4-methyl-	11.36	C_7_H_9_NO_2_	41	67.1	0.01	29995	+	66	–
acetamide, *N*-(2-ethoxy-3,6-dihydro-6-methyl-2*H*-pyran-3-yl)-	13.42	C_10_H_17_NO_3_	32	125.13	0.01	558082	+	92	–
butanoic acid	15.48	C_4_H_8_O_2_	35	60.06	0.02	264	–	0	+
2-imidazolidinone	16.33	C_3_H_16_N_2_O	16	86.07	0.01	8453	+	118	–
2-heptanone	16.98	C_7_H_14_O	87	58.08	0.04	8051	–	0	+
*n*-decanoic acid	17.74	C_10_H_20_O_2_	149	60.06	0.05	2969	–	0	+
hexanoic acid	18.72	C_6_H_12_O_2_	44	60.06	0.03	8892	–	0	+
2-pentadecanone	19.04	C_15_H_30_O	97	58.09	0.06	61303	–	0	+
*n*-hexadecanoic acid	19.57	C_16_H_32_O_2_	3776	73.07	7.57	985	–	0	+
nonanamide	19.67	C_9_H_19_NO	200	59.08	0.23	70709	+	0	–
nonanoic acid	20.38	C_9_H_18_O_2_	87	73.07	0.12	8158	–	0	–
9,12-octadecadienoic acid (*Z*,*Z*)-	21.08	C_18_H_32_O_2_	665	67.1	4.20	5280450	–	0	+
*trans*-13-octadecenoic acid	21.12	C_18_H_34_O_2_	1056	55.1	16.04	6161490	–	0	+
oleic acid	21.31	C_18_H_34_O_2_	458	55.1	0.93	445639	–	0	+
nonanamide	21.34	C_9_H_19_NO	274	59.08	0.41	70709	+	0	–
nonanamide	21.51	C_9_H_19_NO	814	59.08	1.06	70709	+	0	–
acetamide, *N*-[(dimethylamino)methylidene]	23.47	C_5_H_10_N_2_O	50	99.07	0.09	567145	+	169	–
9-octadecenamide, (*Z*)-	23.79	C_18_H_35_NO	1360	59.08	4.67	5283387	+	0	+
13-docosenamide, (*Z*)-	23.88	C_22_H_43_NO	115	59.08	0.66	5365371	+	0	–

a (+) = Present (compound matches SMARTS rule or belongs to cytotoxic cluster); (−) = Absent (no SMARTS match and not part of cytotoxic cluster).

The cytotoxic chemical space of *C. siliqua* was dominated by medium- to long-chain fatty acids and ketones, including hexanoic, decanoic, *n*-hexadecanoic, linoleic, and trans-13-octadecenoic acids. These lipidic metabolites are well-documented for inducing mitochondrial dysfunction, ROS generation, and membrane destabilization, with cytotoxic potency strongly influenced by chain length and unsaturation.^80,81^ Alongside these lipids, the extract also contained several electrophilic ketones, including 2-nonanone, 2-heptanone, and 2-pentadecanone. Compounds with α-keto or enone groups often show cytotoxicity because they can react with protein thiols, inhibit key metabolic enzymes, and promote oxidative stress.^82^ There were also nitrogen-containing heterocycles such as pyrrolidine-1-acetonitrile, succinimide, and imidazolidinone derivatives. These molecules have been linked to effects like cell-cycle disruption, DNA–protein cross-linking, and shifts in cellular redox balance.^83–85^ Also, fatty amides such as nonanamide, oleamide, and 13-docosenamide added another layer of activity. These amides are reported to influence apoptosis, calcium signaling, and membrane fluidity and have been found in other cytotoxic plant extracts.^86,87^ Therefore, the cytotoxic profile reflects the interplay of membrane-active fatty acids, electrophilic ketones, and bioactive nitrogen heterocycles and amides, indicating multiple converging mechanisms and providing a strong basis for prioritizing antiproliferative candidates.

#### 3.6.1 Cytotoxic Scaffold Analysis

Scaffold analysis of the cytotoxic data set showed six distinct structural frameworks (Figure 16). Scaffold 1 occurred twice and represented the only repeating core, while the remaining scaffolds (Scaffolds 2–6) each appeared once. These frameworks were mainly composed of medium- to long-chain fatty acids, ketones, and small nitrogen-containing heterocycles, all commonly associated with cytotoxic mechanisms in plant metabolites. The cytotoxic space is defined by one dominant scaffold supported by several single-occurrence frameworks that add complementary chemical diversity.

**Figure 16:**
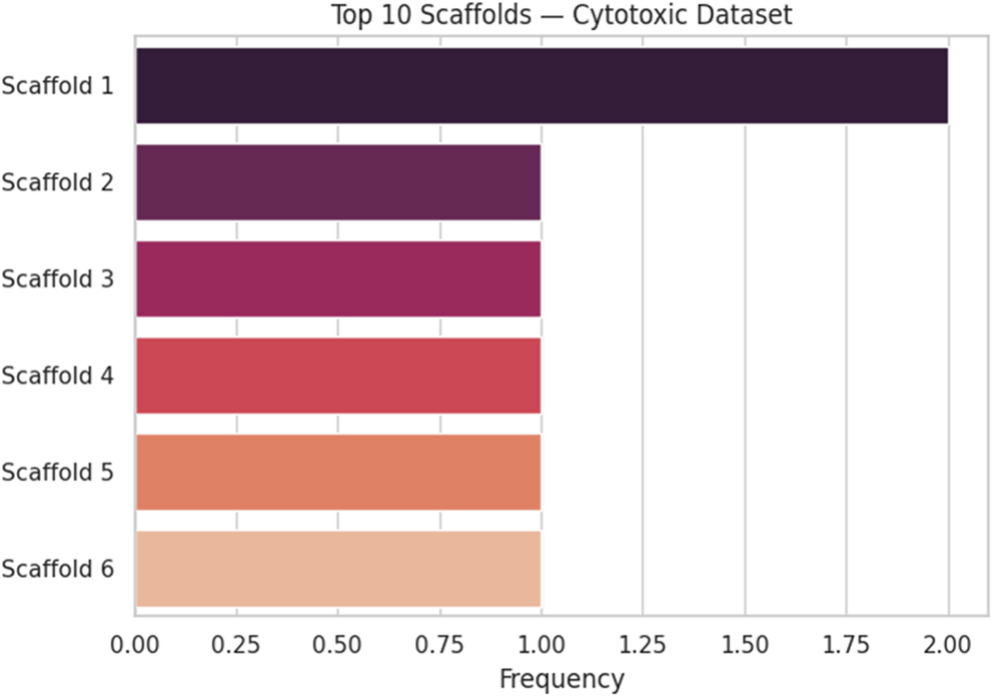
Distribution of the top 10 scaffolds in the cytotoxic data set.

Figure 17 shows the six scaffolds identified in the cytotoxic set. Only Scaffold 1 appeared more than once, representing a small lactam-dione ring, a motif often linked to reactive or mildly electrophilic behavior. The remaining scaffolds (2–6) occurred once each. Most of them are small oxygen- or nitrogen-containing rings, including imides, lactone-like motifs, and an oxygenated cyclohexene. These types of heterocycles are commonly associated with cytotoxic effects because of their ability to interact with cellular targets through mild electrophilicity or redox activity.

**Figure 17:**
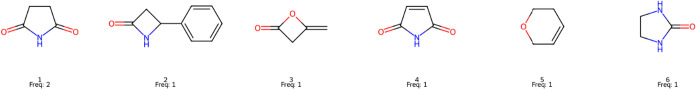
Six distinct scaffolds identified in the cytotoxic-associated GC–MS data set of *C. siliqua* L. extract.

Therefore, the cytotoxic subset is small but chemically focused, dominated by reactive heterocycles rather than terpene-type rings, setting it apart from antioxidant and antimicrobial profiles.

The six Bemis–Murcko scaffolds identified within the cytotoxic subset were each illustrated using one representative compound to highlight their underlying chemical features. Scaffold 1 included the imide-type structures pyrrolidine-1-acetonitrile, 2,5-dioxo- and succinimide, both simple rings commonly associated with stress- and apoptosis-related effects. Scaffold 2 was represented by the β-lactam 2-azetidinone, 1-*tert*-butyl-3,3-dimethyl-4-phenyl-, a small strained ring often linked to reactive, cytotoxic behavior.

Scaffold 3, shown by 2-oxetanone, 4-methylene-, captured a compact oxetanone ring. Scaffold 4 was represented by 1*H*-pyrrole-2,5-dione, 3-ethyl-4-methyl-, a small dione-containing heterocycle. Scaffold 5 was illustrated by the oxygenated acetamide–pyran compound acetamide, *N*-(2-ethoxy-3,6-dihydro-6-methyl-2H-pyran-3-yl)-. Finally, Scaffold 6 was represented by 2-imidazolidinone, a simple imidazolidinone ring. These six representative structures are shown in Figure 18.

**Figure 18:**
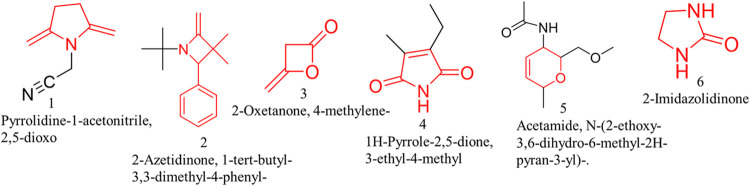
Chemical structures of the top representative cytotoxic-associated compounds, each annotated with its corresponding identified scaffold highlighted in red. Structures were generated using ChemDraw.

#### 3.6.2 Molecular Docking Studies of Cytotoxic-Associated Compounds

Binding mode analysis provides insight into the interaction of cytotoxic-associated compounds (Figure 18) with epidermal growth factor receptor (EGFR) (PDB: 7ZYM), targeting the EGFR-T790M/C797S binding site. This binding site is responsible for ATP binding and phosphorylation, which activates downstream PI3K/AKT and MAPK/ERK pathways involved in cell proliferation and survival.^88^

For the molecular interaction of cytotoxic-associated compounds and the standard reference with mutated EGFR (PDB ID: 7ZYM), doxorubicin displayed the highest binding affinity (−8.2 kcal/mol, *K*_*i*_ = 0.974 μM), followed by 2-azetidinone, 1-*tert*-butyl-3,3-dimethyl-4-phenyl (−7.0 kcal/mol, *K*_*i*_ = 7.336 μM) and acetamide, *N*-(2-ethoxy-3,6-dihydro-6-methyl-2*H*-pyran-3-yl)- (−5.3 kcal/mol, *K*_*i*_ = 130.181 μM) (Table 7). Although the reference compound demonstrated stronger binding, the identified compounds still showed reasonable interactions with the target protein. Key interactions were observed with important residues such as LYS745, MET790, MET793, SER797, and ASP800, which are known to play a role in EGFR mutant selectivity.^40^ Notably, acetamide, *N*-(2-ethoxy-3,6-dihydro-6-methyl-2*H*-pyran-3-yl)- formed a hydrogen bond with SER797 (2.98 Å), while both this compound and 2-azetidinone derivatives exhibited π–sigma interactions with LEU718 (Figure 19). Therefore, the presence of hydrogen bonding and hydrophobic interactions within the active site suggests stable ligand binding and indicates that these compounds may contribute to the observed cytotoxic activity.

**Figure 19:**
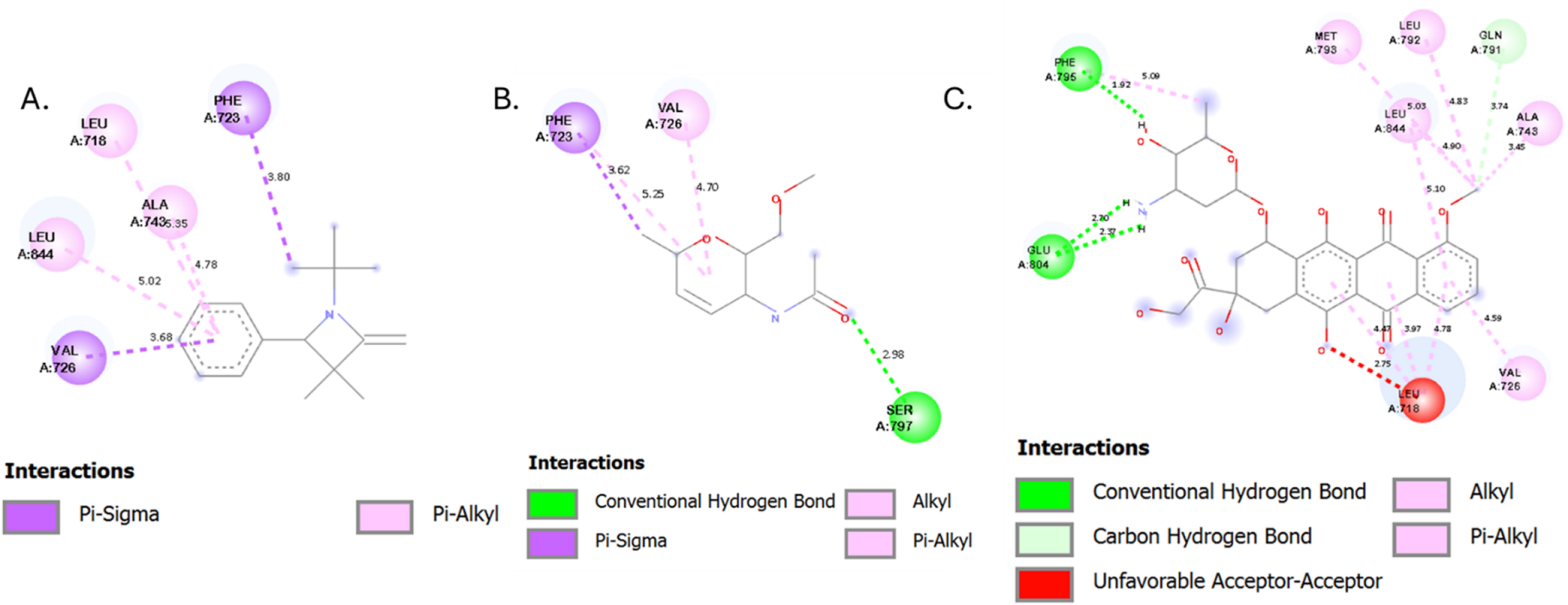
2D interaction of (A) 2-azetidinone, 1-*tert*-butyl-3,3-dimethyl-4-phenyl-, (B) acetamide, *N*-(2-ethoxy-3,6-dihydro-6-methyl-2*H*-pyran-3-yl)-, and the standard drug (C) doxorubicin with EGFR (PDB: 7ZYM).

**Table 7:** Molecular Docking Results for the Top Cytotoxic-Associated Compounds and Reference Standard Drugs against EGFR Target

ligand	binding energy (kcal/mol) (7ZYM)	inhibition constant (μM)
pyrrolidine-1-acetonitrile, 2,5-dioxo-	–4.8	303.003
2-azetidinone, 1-*tert*-butyl-3,3-dimethyl-4-phenyl-	–7.0	7.336
2-oxetanone, 4-methylene-,	–3.4	3244.482
acetamide, *N*-(2-ethoxy-3,6-dihydro-6-methyl-2*H*-pyran-3-yl)-	–5.3	130.181
1*H*-pyrrole-2,5-dione, 3-ethyl-4-methyl	–5.1	182.147
2-imidazolidinone	–3.3	3849.648
doxorubicin	–8.2	0.974

#### 3.6.3 Cytotoxicity Activity of *C. siliqua* L. Pod Extract

Breast cancer is the most common cancer among women and ranks second in mortality rates, while the incidence of colorectal cancer is increasing globally, placing a significant burden on healthcare systems. An effective anticancer agent aims to target cancer cells while minimizing toxicity to healthy cells.^89^ In this context, the toxicity of the hexane pod extract from *C. siliqua* was evaluated against normal (Hek293) cells, breast cancer (MCF-7), and colorectal cancer (HCT-116) cell lines, using the MTT assay. As shown in Table 8, the results indicated that the *C. siliqua* extract displayed superior anticancer activity against HCT-116 cells, with an IC_50_ value of 7.66 ± 0.81 μg/mL, compared to MCF-7 cells, which showed an IC_50_ of 14.3 ± 1.13 μg/mL. Notably, the extract exhibited lower toxicity to normal Hek293 cells, achieving an IC_50_ value of 27.1 ± 1.34 μg/mL. In contrast, doxorubicin, a widely used nonselective anticancer drug, demonstrated significantly higher toxicity across all cell lines, with IC_50_ values of 0.54 ± 0.059 μg/mL, 0.46 ± 0.057 μg/mL, and 0.260 ± 0.30 μg/mL for HCT-116, Hek293, and MCF-7, respectively. At a concentration of 0.1%, DMSO demonstrated no noteworthy effect on the viability of the cell lines in comparison to the untreated media controls. This finding verifies that DMSO does not influence the biological activity of the compounds under investigation. The differences in IC_50_ values between the *C. siliqua* extract and the doxorubicin control were statistically significant (*p* < 0.0001). The selectivity index (SI) calculation for the extract revealed values of 3.5 and 2, indicating that the hexane extract exhibits greater toxicity toward MCF-7 and HCT-116 cancer cell lines compared to normal HEK293 cells (Figure 20). Previous research has shown that less-polar compounds, such as sitosterol, stigmasterol, and lupeol present in the extract, may contribute to its cytotoxic effects against cancer cells.^90^

**Figure 20:**
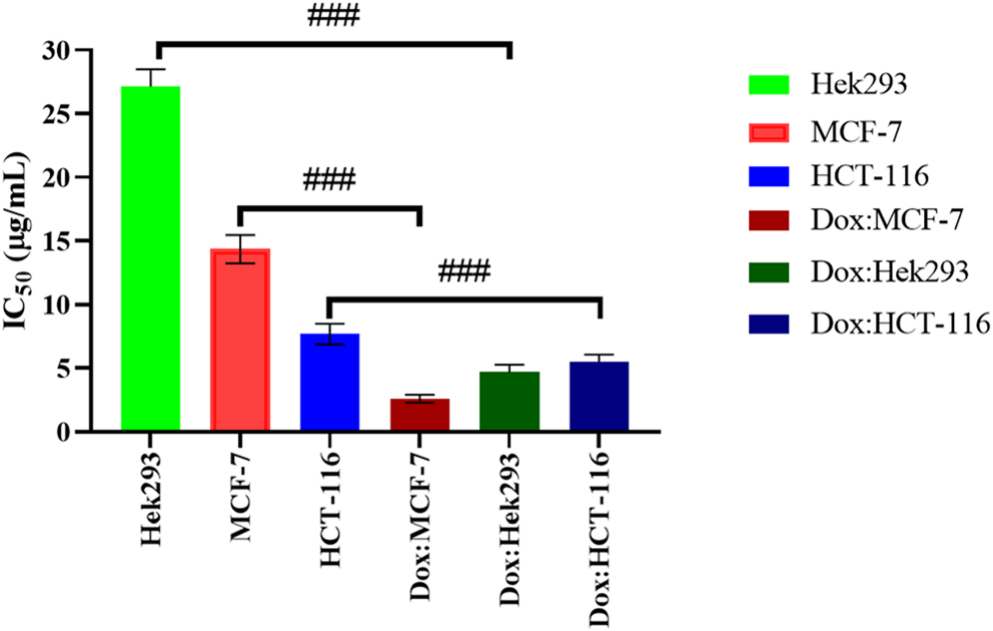
Bar graph demonstrating the cytotoxic effects of *C. siliqua* on normal cells (HEK293) and cancer cell lines (MCF-7 and HCT-116) determined via a cell viability assay. Values are expressed as the mean of triplicate analyses (*n* = 3) ± standard deviation. (###) Represents the statistical difference (*p* < 0.0001) when the sample is compared to the control.

**Table 8:** Cytotoxicity MIC Values of *C. siliqua* Methanol and Hexane Extracts against Hek293, MCF-7, and HCT-116 Cell Lines^a^

	IC_50_ (μg/mL)		
extracts	HCT-116	Hek293	MCF-7
*C. siliqua* (Hex)	7.66 ± 0.81	27.1 ± 1.34	14.3 ± 1.13
doxorubicin	0.54 ± 0.59	0.46 ± 0.57	0.26 ± 0.30

a Values are expressed as the mean of triplicate analyses (*n* = 3) ± standard deviation.

Therefore, the six representative compounds selected from the dominant cytotoxic scaffolds (Figure 18) reflect the core structural features most likely underlying the selective cytotoxic effects observed in the MTT assay. The strong activity against HCT-116 and MCF-7 cells, together with the lower toxicity toward normal HEK293 cells, aligns with GC–MS-identified constituents belonging to reactive imide rings, β-lactams (2-azetidinone, 1-*tert*-butyl-3,3-dimethyl-4-phenyl), oxetanones, and dione-bearing heterocycles. These strained-ring systems are chemical motifs frequently associated with apoptosis induction, oxidative stress-related mechanisms, and growth-inhibitory behavior in cancer cells.^91,92^ Their presence within the extract provides a structural explanation for the cytotoxic and antiproliferative activity response, which reinforces the link between the identified GC–MS compounds and the observed bioassay.

#### 3.6.4 H_2_DCF-DA ROS Detection

Intracellular ROS-scavenging activity was evaluated using the H_2_DCF-DA assay to provide mechanistic insight into the oxidative stress-related activity of *Ceratonia siliqua* extract. Based on cytotoxicity profiling, a nontoxic concentration below IC_50_ (5 μg/mL) was selected to assess the inhibitory effect of LPS-induced ROS production. One-way ANOVA and Tukey’s multiple comparison test were used to study the effects of the extract and LPS on ROS levels (Figure 21). Hek293, HCT-116, and MCF-7 cell lines had a high ROS level from LPS stimulation as lipopolysaccharide is known to activate oxidative stress pathways via NF-κB and MAPK signaling pathways (NF-κB pathway; MAPK pathway).^93^ *C. siliqua* hexane extract was added to the LPS-treated cells, and a 53% moderate inhibition was observed against Hek293 cells; on the other hand, the extract exhibited poor ROS inhibitory activity of 40% and 43% on HCT-116 and MCF-7 cells, respectively. The low antioxidant activity of hexane extract may be attributed to the nonpolar content. It is well-known that polar solvents such as methanol and ethanol extract phenolic acids and flavonoids and are the most important elements in the antioxidant and free-radical scavenging. In contrast, nonpolar solvents like hexane mainly extract lipophilic compounds such as fatty acids, sterols, and terpenoids, which are less direct scavenging agents.^94^ Phenolics such as gallic acid, catechins, and quercetin derivatives found in *C. siliqua* are known to have an antioxidant effect through donation of hydrogen atoms and electron transfer.^95^ The low efficacy reported in the hexane extract may therefore reflect low abundance of these bioactive compounds. The lack of aqueous solubility and poor dispersion of lipophilic compounds in cell-based assay systems also can limit cellular uptake and bioavailability, which may further reduce their antioxidant activity.^96,97^

**Figure 21:**
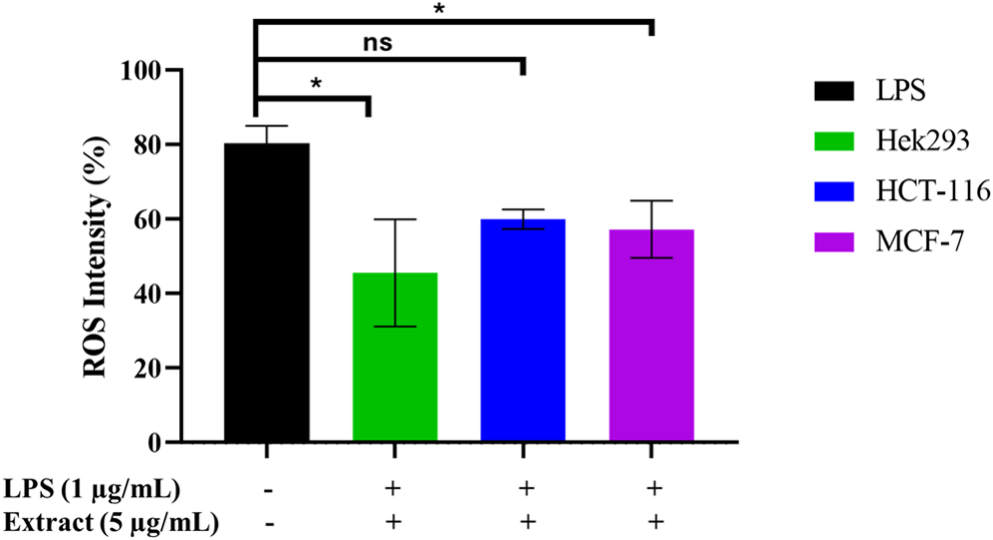
ROS fluorescence intensity in Hek293, HCT-116, and MCF-7 cells treated with *C. siliqua* extract and LPS to trigger inflammation is presented with values expressed as the mean of duplicate analyses (*n* = 2) ± standard deviation. Ns = not significant and * indicates statistical differences among the treatments (*p* < 0.0001).

Therefore, the partial modulation of ROS levels suggests that the cytotoxicity and antioxidant activity of the extract may not be solely dependent on direct radical scavenging. When considered alongside the molecular docking results, which demonstrated favorable interactions of GC–MS-identified compounds with key targets such as KEAP1 and EGFR, these findings support a multitarget mechanism involving both oxidative stress modulation and direct protein interaction. Thus, while the extract exhibited moderate ROS inhibitory activity, the combined evidence from docking and cytotoxicity assays indicates that individual compounds may contribute to the observed biological effects through complementary mechanisms, including redox regulation and interference with cell signaling pathways.

### 3.7 Scaffold Diversity and Cross-Bioactivity Overlap

The three GC–MS activity-based data sets showed clear differences in scaffold diversity and structural dispersion (Table 9). The antimicrobial set was the most scaffold-diverse, with 17 unique scaffolds across 20 molecules (*n*_s_/*n*_s_ = 0.85) and a high proportion of singletons (*n*_ss_/*n*_ss_ = 0.70). Its Shannon entropy (4.02) confirmed a broad and evenly distributed scaffold landscape. In contrast, the antioxidant data set exhibited moderate scaffold diversity (*N*_s_/*N* = 0.42) but a high number of cyclic skeletons relative to size (*N*_csk_/*N* = 0.94), indicating a structurally varied but more scaffold-constrained space. The cytotoxic group, although the smallest, showed the highest proportional scaffold diversity (0.86) because nearly every compound introduced a distinct scaffold. Its entropy (2.52) reflects this small and heterogeneous chemical set.

**Table 9:** Scaffold Diversity Metrics for the Antioxidant, Antimicrobial, and Cytotoxic Data Sets

data set	molecules (*N*)	unique scaffolds (*N*_s_)	singleton scaffolds (*N*_ss_)	cyclic skeletons (*N*_csk_)	*N*_s_/*N*	*N*_ss_/*N*	*N*_csk_/*N*	scaffold diversity (N/M)	singleton diversity (*N*_sing_/*M*)	Shannon entropy
antioxidant	31	13	9	29	0.419	0.290	0.935	0.419	0.290	2.781
antimicrobial	20	17	14	18	0.850	0.700	0.900	0.850	0.700	4.022
cytotoxic	7	6	5	7	0.857	0.714	1.000	0.857	0.714	2.522

Across the data sets, most scaffolds were unique to a single activity class. From Figure 22, the strongest overlap was between the antimicrobial and antioxidant groups, which shared four scaffolds. These included a cyclohexenyl framework, an oxygenated cyclohexanone scaffold, a bicyclic monoterpenoid core, and a simple cyclohexyl ring system. The compounds linking these groups were terpinen-4-ol and trans-3(10)-caren-2-ol, both well-known monoterpenoids commonly associated with antimicrobial and antioxidant activities. The antimicrobial and cytotoxic sets shared only one scaffold, which is a nitrogen-containing cyclopentene with two carbonyl groups, a structure typical of imide-like cytotoxic motifs. This overlap was represented by 1*H*-pyrrole-2,5-dione, 3-ethyl-4-methyl-, a small electrophilic heterocycle often linked to cytotoxic behavior. No scaffold appeared across all three activity classes, highlighting that antioxidant, antimicrobial, and cytotoxic actions in the GC–MS data set arise from mostly distinct structural families rather than a single shared chemical core.

**Figure 22:**
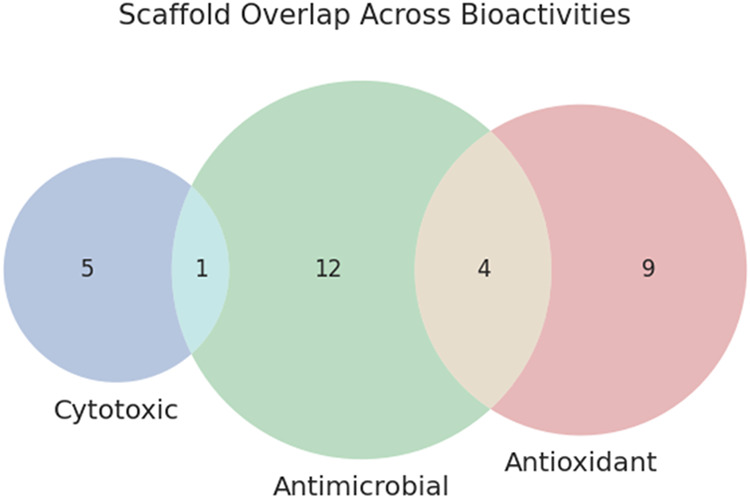
Venn diagram shows scaffold overlaps across antioxidant, antimicrobial, and cytotoxic data sets.

## 4 Conclusion

This research provides an integrated chemical and biological evaluation of the hexane extract from *Ceratonia siliqua* L. pods, using scaffold-based drug analysis to connect the core structural frameworks of the GC–MS-identified compounds to antioxidant, antimicrobial, and cytotoxic activities. Indeed, the extract demonstrated measurable antioxidant capacity, moderate antibacterial action against *S. aureus*, and notable selectivity toward cancer cells, especially HCT-116, while being less toxic to normal HEK293 cells. The modulation of LPS-induced ROS levels in Hek293, HCT-116, and MCF-7 cells indicates that the hexane extract of *C. siliqua* exhibits moderate to low oxidative stress inhibitory activity. The observed effect may be attributed to the predominance of less-polar compounds in the extract, which can influence cellular uptake and overall bioavailability.

Linking these observed biological activities to the identified dominant Bemis–Murcko scaffolds enabled the complex mixture of the plant extract to be reduced into a set of core structural frameworks most likely responsible for each biological response. Phenolic and terpenoid scaffolds corresponded with the antioxidant behavior, monoterpenoid, and cyclohexane/cyclohexene-based motifs also aligned with the antimicrobial activity, and last, several small heterocyclic and imide-containing scaffolds matched the cytotoxic trends. Together, these structural motifs, alongside the top GC–MS-identified compounds that map onto them, represent the components of *C. siliqua* L. with the strongest potential for future pharmacological development. To further support these structure–activity relationships, molecular docking provided mechanistic insight into the interaction of GC–MS-identified compounds with key biological targets, including KEAP1, *S. aureus* DHFR, and EGFR. The observed binding affinities and interaction patterns suggest that individual compounds may contribute to the overall biological effects of the extract, with some compounds exhibiting stronger interactions that may play a more prominent role.

Overall, the findings position *C. siliqua* L. not just as a source of bioactive compounds but as a reservoir of promising scaffold templates that merit further isolation and rational optimization within natural-product research in drug discovery.

## Supplementary Material



## Data Availability

The Python scripts used for scaffold-based drug analysis, including the curated data sets, scaffold diversity assessment, cross-bioactivity overlap, and molecular structure processing, are available in the public repository: DOI: 10.5281/zenodo.18734106. The GC–MS data set is provided in CSV format, along with the corresponding TIC and AIC chromatograms. The code for activity-driven compound selection, data set expansion, and Bemis–Murcko scaffold generation is provided as machine-readable. ipynb files and is included within the same repository. All other relevant data generated or analyzed during this study are fully presented within this paper.

## Notes

Cluster summary of GC–MS-identified compounds, showing cluster size, intra- and intercluster similarity values, and corresponding homogeneity scores (Table S1) (PDF)
