# Supplementary material for: Chemical Profiling and Scaffold-Based Drug-Discovery Analysis of Bioactive Compounds from Ceratonia siliqua L. with Computational and Biological Validation
Source: J Chem Inf Model. 2026 May 28;66(12):7065–87. doi: 10.1021/acs.jcim.6c00748 (PMC13292205; doi:10.1021/acs.jcim.6c00748)
Supplement: Supplementary file 1 [file ci6c00748_si_001.pdf]

Chemical Profiling and Scaffold-Based Drug Discovery Analysis of Bioactive Compounds from *Ceratonia siliqua* L. with Computational and Biological Validation.

<u>Authors</u>

Deli-Bright N.T. Oku[a], Garland Kgosi More[b]*, Yannick Nuapia[c], Ramakwala Christinah Chokwe[a]*

dbntoku@gmail.com, moregk@unisa.ac.za*, yannick.nuapia@ul.ac.za, chokwrc@unisa.ac.za*

[a]*Department of Chemistry, The Science Campus, College of Science Engineering and Technology, University of South Africa, Corner Christiaan de Wet Road and Pioneer Avenue, Florida Park, Roodepoort 1709, South Africa*

[b]*College of Agriculture and Environmental Sciences, CAES Laboratories, University of South Africa, Private Bag X6, Florida, Johannesburg, 1710, South Africa.*

[c]*Department of Pharmacy, Turfloop Campus, School of Healthcare Science, University of Limpopo, Polokwane 0727, South Africa.*

Table S1. *Summary of structural clusters derived from GC–MS-identified compounds of Ceratonia siliqua L. The table reports the number of compounds per cluster, mean intra-cluster similarity, mean inter-cluster similarity, and homogeneity scores used to assess structural cohesion across various clustures.*

**Cluster_ID**	**Num_Compounds**	**Intra_Similarity**	**Inter_Similarity**	**Homogeneity**
22	2	1.0	0.083	11.98
19	2	1.0	0.088	11.29
6	5	1.0	0.047	21.09
4	5	1.0	0.150	6.655
23	2	1.0	0.098	10.13
9	3	1.0	0.038	26.37
24	2	1.0	0.115	8.708
10	3	1.0	0.045	22.43
12	3	1.0	0.117	8.507
21	2	1.0	0.094	10.67
13	2	0.85	0.147	5.775
18	2	0.783	0.133	5.889
16	2	0.76	0.126	6.015
14	2	0.738	0.122	6.033
3	5	0.727	0.155	4.706
17	2	0.7	0.088	7.980
15	2	0.7	0.097	7.239
25	2	0.7	0.138	5.067

5	5	0.679	0.132	5.148
11	3	0.672	0.151	4.444
8	4	0.658	0.120	5.468
7	4	0.642	0.099	6.436
20	2	0.613	0.112	5.453
0	19	0.595	0.150	3.963
1	15	0.561	0.156	3.596
2	7	0.518	0.159	3.239
